# A Foundation Model for Cell Segmentation

**DOI:** 10.1101/2023.11.17.567630

**Published:** 2024-03-07

**Authors:** Uriah Israel, Markus Marks, Rohit Dilip, Qilin Li, Changhua Yu, Emily Laubscher, Shenyi Li, Morgan Schwartz, Elora Pradhan, Ada Ates, Martin Abt, Caitlin Brown, Edward Pao, Alexander Pearson-Goulart, Pietro Perona, Georgia Gkioxari, Ross Barnowski, Yisong Yue, David Van Valen

**Affiliations:** 1 Division of Biology and Biological Engineering, Caltech.; 2 Division of Engineering and Applied Science, Caltech.; 3 Division of Computing and Mathematical Science, Caltech.; 4 Division of Chemistry and Chemical Engineering, Caltech.; 5 Howard Hughes Medical Institute.

**Keywords:** cell segmentation, object detection, deep learning, foundation model

## Abstract

Cells are a fundamental unit of biological organization, and identifying them in imaging data – cell segmentation – is a critical task for various cellular imaging experiments. While deep learning methods have led to substantial progress on this problem, most models in use are specialist models that work well for specific domains. Methods that have learned the general notion of “what is a cell” and can identify them across different domains of cellular imaging data have proven elusive. In this work, we present CellSAM, a foundation model for cell segmentation that generalizes across diverse cellular imaging data. CellSAM builds on top of the Segment Anything Model (SAM) by developing a prompt engineering approach for mask generation. We train an object detector, CellFinder, to automatically detect cells and prompt SAM to generate segmentations. We show that this approach allows a single model to achieve human-level performance for segmenting images of mammalian cells (in tissues and cell culture), yeast, and bacteria collected across various imaging modalities. We show that CellSAM has strong zero-shot performance and can be improved with a few examples via few-shot learning. We also show that CellSAM can unify bioimaging analysis workflows such as spatial transcriptomics and cell tracking. A deployed version of CellSAM is available at https://cellsam.deepcell.org/.

## 1 Introduction

Accurate cell segmentation is crucial for quantitative analysis and interpretation of various cellular imaging experiments. Modern spatial genomics assays can produce data on the location and abundance of 10^2^ protein species and 10^3^ RNA species simultaneously in living and fixed tissues ^1–5^. These data shed light on the biology of healthy and diseased tissues but are challenging to interpret. Cell segmentation enables these data to be converted to interpretable tissue maps of protein localization and transcript abundances. Similarly, live-cell imaging provides insight into dynamic phenomena in bacterial and mammalian cell biology. Mechanistic insights into critical phenomena such as the mechanical behavior of the bacterial cell wall ^6,7^, information transmission in cell signaling pathways ^8–11^, heterogeneity in immune cell behavior during immunotherapy ^12^, and the morphodynamics of development^13^ have been gained by analyzing live-cell imaging data. Cell segmentation is also a key challenge for these experiments, as cells must be segmented and tracked to create temporally consistent records of cell behavior that can be queried at scale. These methods have seen use in a number of systems, including mammalian cells in tissues ^5^ and cell culture ^14,15^, bacterial cells ^16–19^, and yeast^20–22^.

Significant progress has been made in recent years on the problem of cell segmentation, primarily driven by advances in deep learning ^23^. Progress in this space has occurred mainly in two distinct directions. The first direction seeks to find deep learning architectures that achieve state-of-the-art performance on cellular imaging tasks. These methods have historically focused on a particular imaging modality (e.g., brightfield imaging) or target (e.g., mammalian tissue) and have difficulty generalizing beyond their intended domain ^24–30^. For example, Mesmer’s ^27^ representation for a cell (cell centroid and boundary) enables good performance in tissue images but would be a poor choice for elongated bacterial cells. Similar trade-offs in representations exist for the current collection of Cellpose models, necessitating the creation of a model zoo ^25^. The second direction is to work on improving labeling methodology. Cell segmentation is a variant of the instance segmentation problem, which requires pixel-level labels for every object in an image. Creating these labels can be expensive ( 10^−2^ USD/label, with hundreds to thousands of labels per image) ^27,31^, which provides an incentive to reduce the marginal cost of labeling. A recent improvement to labeling methodology has been human-in-the-loop labeling, where labelers correct model errors rather than produce labels from scratch ^25,27,32^. Further reductions in labeling costs can increase the amount of labeled imaging data by orders of magnitude.

Recent work in machine learning on foundation models holds promise for providing a complete solution. Foundation models are large deep neural network models (typically transformers ^33^) trained on a large amount of data in a self-supervised fashion with supervised fine-tuning on one or several tasks ^34^. Foundation models include the GPT^35,36^ family of models, which have proven transformative for natural language processing ^34^. The GPT and BERT families of models have recently seen use for biological sequences ^37–41^. These successes have inspired similar efforts in computer vision. The Vision Transformer ^42^ (ViT) was introduced in 2020 and has since been used as the basis architecture for a collection of vision foundation models ^43–47^. One recent foundation model well suited to cellular image analysis needs is the Segment Anything Model (SAM) ^48^. This model uses a Vision Transformer to extract information-rich features from raw images. These features are then directed to a module that generates instance masks based on user provided prompts, which can be either spatial (e.g., an object centroid or bounding box) or semantic (e.g., an object’s visual description). Notably, the promptable nature of SAM enabled scalable dataset construction, as preliminary versions of SAM allowed labelers to generate accurate instance masks with 1–2 clicks. The final version of SAM was trained on a dataset of 11 million images containing over 1 billion masks, and demonstrated strong performance on various zero-shot learning tasks. Recent work has attempted to apply SAM to problems in biological and medical imaging, including medical image segmentation ^49–51^, lesion detection in dermatological images ^52,53^, nuclear segmentation in H&E images ^54,55^, and cellular image data for use in the Napari software package ^56^.

While promising, these studies reported challenges adapting SAM to these new use cases ^49,56^. These challenges include reduced performance and uncertain boundaries when transitioning from natural to medical images. Cellular images contain additional complications – they can involve different imaging modalities (e.g., phase microscopy vs. fluorescence microscopy), thousands of objects in a field of view (as opposed to dozens in a natural image), and uncertain and noisy boundaries (artifacts of projecting 3D objects into a 2D plane) ^56^. In addition to these challenges, SAM’s default prompting strategy does not allow for accurate inference on cellular images. Currently, the automated prompting of SAM uses a uniform grid of points to generate masks, an approach poorly suited to cellular images given the wide variation of cell densities. More precise prompting (e.g., a bounding box or mask) requires prior knowledge of cell locations. This creates a weak tautology - SAM can find the cells provided it knows where they are. This limitation makes it challenging for SAM to serve as a foundation model for cell segmentation since it still requires substantial human input for inference. A solution to this problem would enable SAM-like models to serve as foundation models and knowledge engines, as they could accelerate the generation of labeled data, learn from them, and make that knowledge accessible to life scientists via inference.

**Fig. 1:**
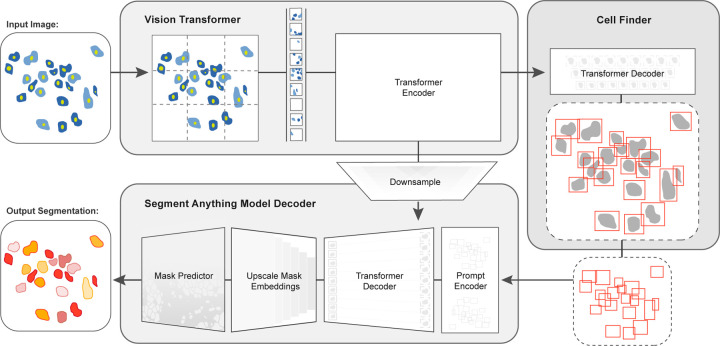
CellSAM: a foundational model for cell segmentation. CellSAM combines SAM’s mask generation and labeling capabilities with an object detection model to achieve automated inference. Input images are divided into regularly sampled patches and passed through a transformer encoder (i.e., a ViT) to generate information-rich image features. These image features are then sent to two downstream modules. The first module, CellFinder, decodes these features into bounding boxes using a transformer-based encoder-decoder pair. The second module combines these image features with prompts to generate masks using SAM’s mask decoder. CellSAM integrates these two modules using the bounding boxes generated by CellFinder as prompts for SAM. CellSAM is trained in two stages, using the pre-trained SAM model weights as a starting point. In the first stage, we train the ViT and the CellFinder model together on the object detection task. This yields an accurate CellFinder but results in a distribution shift between the ViT and SAM’s mask decoder. The second stage closes this gap by fixing the ViT and SAM mask decoder weights and fine-tuning the remainder of the SAM model (i.e., the model neck) using ground truth bounding boxes and segmentation labels.

In this work, we developed CellSAM, a foundation model for cell segmentation (Fig. 1). CellSAM extends the SAM methodology to perform automated cellular instance segmentation. To achieve this, we first assembled a comprehensive dataset for cell segmentation spanning five broad data archetypes: tissue, cell culture, yeast, H&E, and bacteria. Critically, we removed data leaks between training and testing data splits to ensure an accurate assessment of model performance. To automate inference with SAM, we took a prompt engineering approach and explored the best ways to prompt SAM to generate high-quality masks. We observed that bounding boxes consistently generated high-quality masks compared to alternative approaches. To facilitate automated inference through prompting, we developed CellFinder, a transformer-based object detector that uses the Anchor DETR framework ^57^. CellSAM and CellFinder share SAM’s ViT backbone for feature extraction; the bounding boxes generated by CellFinder are then used as prompts for SAM, enumerating masks for all the cells in an image. We trained CellSAM on a large, diverse corpus of cellular imaging data, enabling it to achieve state-of-the-art (SOTA) performance across ten datasets. We also evaluated CellSAM’s zero-shot performance using a held-out dataset, LIVECell ^58^, demonstrating that it substantially outperforms existing methods for zero-shot segmentation. A deployed version of CellSAM is available at https://cellsam.deepcell.org.

## 2 Results

### 2.1 Construction of a dataset for general cell segmentation

A significant challenge with existing cellular segmentation methods is their inability to generalize across cellular targets, imaging modalities, and cell morphologies. To address this, we curated a dataset from the literature containing 2D images from a diverse range of targets (mammalian cells in tissues and adherent cell culture, yeast cells, bacterial cells, and mammalian cell nuclei) and imaging modalities (fluorescence, brightfield, phase contrast, and mass cytometry imaging).

**Fig. 2:**
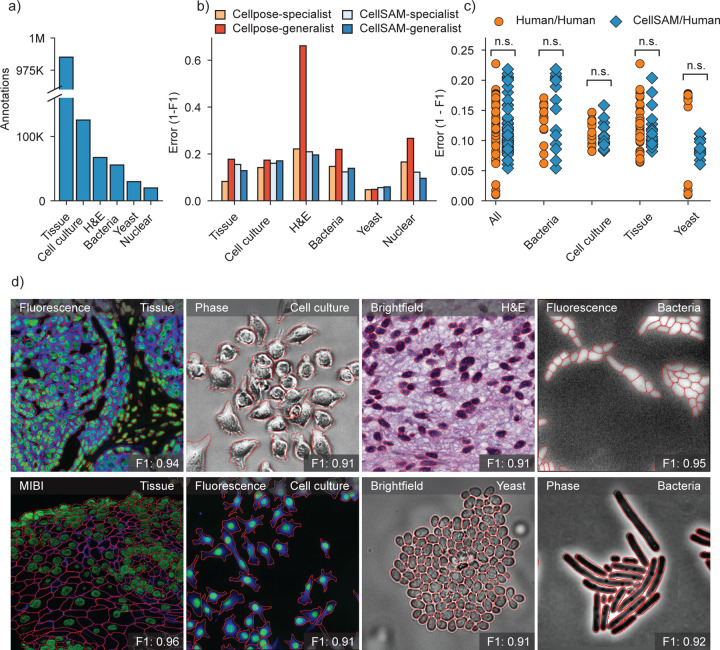
CellSAM is a strong generalist model for cell segmentation. a) For training and evaluating CellSAM, we curated a diverse cell segmentation dataset from the literature. The number of annotated cells is given for each data type. Nuclear refers to a heterogeneous dataset (DSB) ^65^ containing nuclear segmentation labels. b) Segmentation performance for CellSAM and Cellpose across different data types. We compared the segmentation error (1-F1) for models that were trained as specialists (i.e., on one dataset) or generalists (i.e., the full dataset). Models were trained for a similar number of steps across all datasets. We observed that CellSAM-generalhad a lower error than Cellpose-general on almost all tested datasets. Furthermore, we observed that generalist training either preserved or improved CellSAM’s performance compared to specialist training. c) Human vs human and CellSAM-general vs human (CS/human) inter-rater performance comparison. A t-test confirms that no statistical difference between CellSAM and human performance exists. d) Qualitative results of CellSAM segmentations for different data and imaging modalities. Predicted segmentations are outlined in red.

Our final dataset consisted of TissueNet ^27^, DeepBacs ^59^, BriFiSeg ^60^, Cellpose ^24,25^, Omnipose ^61,62^, YeastNet ^63^, YeaZ ^64^, the 2018 Kaggle Data Science Bowl dataset (DSB) ^65^, a collection of H&E datasets ^66–72^, and an internally collected dataset of phase microscopy images across eight mammalian cell lines (Phase400). We group these datasets into six types for evaluation: Tissue, Cell Culture, H&E, Bacteria, and Yeast. As the DSB ^65^ comprises cell nuclei that span several of these types, we evaluate it separately and refer to it as Nuclear, making a total of six categories for evaluation. While our method focuses on whole-cell segmentation, we included DSB ^65^ because cell nuclei are often used as a surrogate when the information necessary for whole-cell segmentation (e.g., cell membrane markers) is absent from an image. Fig. 2a shows the number of annotations per evaluation type. Finally, we used a held-out dataset LIVECell ^58^ to evaluate CellSAM’s zero-shot performance. This dataset was curated to remove low-quality images, as well as images that did not contain sufficient information about the boundaries of closely packed cells. A detailed description of data sources and pre-processing steps can be found in Appendix A.

### 2.2 Bounding boxes are accurate prompts for cell segmentation with SAM

For accurate inference, SAM needs to be provided with approximate information about the location of cells in the form of prompts. To better engineer prompts, we first assessed SAM’s ability to generate masks with provided prompts derived from ground truth labels - either point prompts (derived from the cell’s center of mass) or bounding box prompts. For these tests, we used the pre-trained model weights that were publicly released ^48^. Our benchmarking revealed that bounding boxes had significantly higher zero-shot performance than point prompting (Fig. S3). However, both approaches struggle to achieve performance standards required for real world use, which we take as an error / 0.2 (Fig. 2c). To improve SAM’s mask generation ability for cellular image data, we explored fine-tuning SAM on our compiled data to help it bridge the gap from natural to cellular images. During these fine-tuning experiments, we observed that fine-tuning all of SAM was unnecessary; instead, we only needed to fine-tune the layers connecting SAM’s ViT to its decoder, the model neck, to achieve good performance (Fig. 1). All other layers can be frozen. Fine-tuning SAM in this fashion led to a model capable of generating high-quality cell masks when prompted by ground truth bounding boxes, as seen in Fig. S3.

### 2.3 CellFinder enables accurate and automated cell segmentation for CellSAM

Because the ground-truth bounding box prompts yield accurate segmentation masks from SAM across various datasets, we sought to develop an object detector that could generate prompts for SAM in an automated fashion. Given that our zero-shot experiments demonstrated that ViT features can form robust internal representations of cellular images, we reasoned that we could build an object detector using the image features generated by SAM’s ViT. Previous work has explored this space and demonstrated that ViT backbones can achieve SOTA performance on natural images ^73,74^. For our object detection module, we use the Anchor DETR^57^ framework with the same ViT backbone as the SAM module; we call this object detection module CellFinder. Anchor DETR is well-suited for object detection in cellular images because it formulates object detection as a set prediction task. This allows it to perform cell segmentation in images with densely packed objects, a common occurrence in cellular imaging data. Alternative bounding box methods (e.g., the R-CNN family) rely on non-maximum suppression ^75,76^, leading to poor performance in this regime. Methods that frame cell segmentation as a dense, pixel-wise prediction task (e.g., Mesmer ^27^, Cellpose ^24^, and Hover-net^29^) assume that each pixel can be uniquely assigned to a single cell and cannot handle overlapping objects.

We train CellSAM in two stages; the full details can be found in the supplement. In the first stage, we train CellFinder on the object detection task. We convert the ground truth cell masks into bounding boxes and train the ViT backbone and the CellFinder module. Once CellFinder is trained, we freeze the model weights of the ViT and fine-tune the SAM module as described above. This accounts for the distribution shifts in the ViT features that occur during the CellFinder training. Once training is complete, we use CellFinder to prompt SAM’s mask decoder. We refer to the collective method as CellSAM; Fig. 1 outlines an image’s full path through CellSAM during inference. We benchmarked CellSAM’s performance using a suite of metrics (Fig. 2b and 2b and Supplemental Fig. S2) and found that it outperformed generalist Cellpose models and was equivalent to specialist Cellpose models trained on comparable datasets. We highlight features of our benchmarking analyses below.

- **CellSAM is a strong generalist model**. Generalization across cell morphologies and imaging datasets has been a significant challenge for deep learning-based cell segmentation algorithms. To evaluate CellSAM’s generalization capabilities, we compared the performance of CellSAM and Cellpose models trained as specialists (i.e., on a single dataset) to generalists (i.e., on all datasets). Consistent with the literature, we observe that Cellpose’s performance degraded when trained as a generalist (Fig. 2c). In contrast, we observed that CellSAMpreserved its performance in the generalist setting. Here we defined two methods to have equivalent performance if the difference in F1 scores was ¡ 0.05, which was the standard deviation of the human-human annotator agreement (Fig. 2c). By this definition, we found the performance of CellSAM-general was equivalent to CellSAM-specific across all data categories and datasets. Moreover, CellSAM-general performed equivalent to Cellpose-specific on five of six data categories and eight of the ten datasets (Fig. 2b and Supplemental Fig. S2), with the exceptions being the DSB and Phase400 datasets. This analysis highlights an essential feature of a foundational model: maintaining performance with increasing data diversity and scale.
- **CellSAM achieves human-level accuracy for generalized cell segmentation.** We use the error (1-F1) to assess the consistency of segmentation predictions and annotator masks across a series of images. We compared the annotations of three experts with each other (human vs. human) and with CellSAM (human vs. CellSAM). We compared annotations across four data categories: mammalian cells in tissue, mammalian cells in cell culture, bacterial cells, and yeast cells. Our analysis revealed no significant differences between these two comparisons, indicating that CellSAM’s outputs are comparable to expert human annotators (Fig. 2f).
- **CellSAM enables fast and accurate labeling** When provided with ground truth bounding boxes, CellSAM achieves high-quality cell masks without any fine-tuning on unseen datasets (Figure S4). Because drawing bounding boxes consumes significantly less time than drawing individual masks, this means CellSAM can be used to quickly generate highly accurate labels, even for out-of-distribution data.
- **CellSAM is a strong zero-shot and few-shot learner**. We used the LIVECell dataset to explore CellSAM’s performance in zero-shot and few-shot settings. We stratified CellSAM’s zero-shot by cell lines present in LIVECell (Supplemental Fig. S4b). We found that while performance varied by cell line, we could recover adequate performance in the few-shot regime (e.g., *A172* ). Supplemental Figures S4 and S5 show that CellSAM can considerably improve its performance with only ten additional fields of views (10^2^ *—* 10^3^ cells) for each cell line. For cell lines with morphologies far from the training data distribution (e.g., *SHSY5Y* ), we found fine-tuning could not recover performance. This may reflect a limitation of bounding boxes as a prompting strategy for SAM models.

### 2.4 CellSAM unifies biological image analysis workflows

**Fig. 3:**
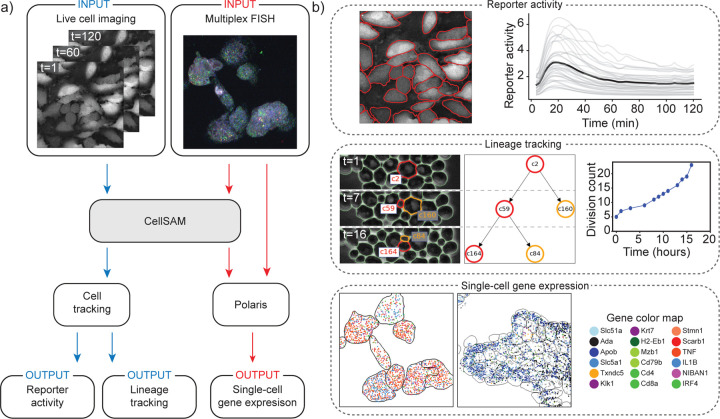
CellSAM unifies biological imaging analysis workflows. Because CellSAM functions across image modalities and cellular targets, it can be immediately applied across bioimaging analysis workflows without requiring task-specific adaptations. (a) We schematically depict how CellSAM fits into the analysis pipeline for live cell imaging and spatial transcriptomics, eliminating the need for different segmentation tools and expanding the scope of possible assays to which these tools can be applied. (b) Application of CellSAM to various biological imaging. (Top) Segmentations from CellSAM are used to track cells ^81^ and quantify fluorescent live-cell reporter activity in cell culture. (Middle) CellSAM segments cells in multiple frames from a video of budding yeast cells. These cells are tracked across frames using a tracking algorithm ^81^ that ensures consistent identities, enabling accurate lineage construction and cell division quantification. (Bottom) Segmentations generated using CellSAM integrate with Polaris ^79^, a spatial transcriptomics analysis pipeline. Because of CellSAM ‘s generalist nature, we can apply this workflow across sample types (e.g., tissue and cell culture) and imaging modalities (e.g., seqFISH and MERFISH). Datasets of cultured macrophage cells (seqFISH) and mouse ileum tissue (MERFISH) ^80^ were used to generate the data in this example. MERFISH segmentations were generated with CellSAM with an image of a nuclear and membrane stain; seqFISH segmentations were generated with CellSAM with a maximum intensity projection image of all spots.

Cell segmentation is a critical component of many spatial biology analysis pipelines; a single foundation model that generalizes across cell morphologies and imaging methods would fill a crucial gap in modern biological workflows by expanding the scope of the data that can be processed. In this section, we demonstrate how CellSAM ‘s generality can diversify the scope of biological imaging analysis pipelines by highlighting two use cases – spatial transcriptomics and cell tracking (Fig.3).

Spatial transcriptomics methods measure single-cell gene expression while retaining the spatial organization of the sample. These experiments (e.g., MERFISH ^77^ and seqFISH ^78^) fluorescently label individual mRNA transcripts; the number of spots for a gene inside a cell corresponds to that gene’s expression level in that cell. These methods enable the investigation of spatial gene expression patterns from the sub-cellular to tissue scales but require accurate cell segmentation to yield meaningful insights. Here, we use CellSAM in combination with Polaris ^79^, a deep learning-enabled analysis pipeline for image-based spatial transcriptomics, to analyze gene expression at the single-cell level in MERFISH ^80^ and seqFISH ^78^ data (Fig. 3). With accurate segmentation, we can assign genes to specific cells (Fig. 3). We note that CellSAM can perform segmentation on either images of nuclear and membrane stains or images derived from the spots themselves (e.g., a maximum intensity projection of all spots). CellSAM’s ability to perform nuclear and whole-cell segmentation for challenging tissue images of dense cells with complex morphologies expands the scope of datasets to which Polaris can be applied.

The dynamics of cellular systems captured by live-cell imaging experiments elucidate various cellular processes such as cell division, morphological transitions, and signal transduction ^32^. The analysis of live-cell imaging data requires segmenting and tracking individual cells throughout whole movies. Here, we use CellSAM in combination with a cell tracking algorithm ^81^ (Fig. 3) in two settings. The first was a live cell imaging experiment with HeLa cells transiently expressing an AMP Kinase reporter ^82^ dosed with 20mM 2-Deoxy-D-glucose, a setup reflective of many experiments exploring cell signaling dynamics ^14^. We imaged, segmented, and tracked the cells over 60 frames or 120 minutes to quantify AMP Kinase activity over time (Fig. 3). The second setting was lineage tracking in budding yeast cells. We again used CellSAM and cell tracking to segment and track cells; we further used a division detection algorithm to count the cumulative number of divisions over time and trace individual cell lineages (Fig. 3). While these use cases span diverse biological image data, their analysis can be simplified into a few key steps. As CellSAM demonstrates, as the algorithms that perform these steps generalize, so too do the entire pipelines.

## 3 Discussion

Cell segmentation is a critical task for cellular imaging experiments. While deep learning methods have made substantial progress in recent years, there remains a need for methods that can generalize across diverse images and further reduce the marginal cost of image labeling. In this work, we sought to meet these needs by developing CellSAM, a foundation model for cell segmentation. Transformer-based methods for cell segmentation are showing promising performance. CellSAM builds on these works by integrating the mask generation capabilities of SAM with transformer-based object detection to empower both scalable image labeling and automated inference. We trained CellSAM on a diverse dataset, and our benchmarking demonstrated that CellSAM achieves human-level performance on generalized cell segmentation. Compared to previous methods, CellSAM preserves its performance when trained on increasingly diverse data, which is essential for a foundational model. We found that CellSAM could be used on novel cell types in a zero-shot setting, and that re-training with few labels could yield a strong boost in performance if needed. Moreover, we demonstrated that CellSAM’s ability to generalize can be extended to entire image analysis pipelines, as illustrated by use cases in spatial transcriptomics and live cell imaging. Given its utility in image labeling and high accuracy during inference, we believe CellSAM is a valuable contribution to the field, both as a tool for spatial biology and as a means to creating the data infrastructure required for cellular imaging’s AI-powered future. To facilitate the former, we have deployed a user interface for CellSAM at https://cellsam.deepcell.org/ that allows for both automated and manual prompting.

The work described here has importance beyond aiding life scientists with cell segmentation. First, foundation models are immensely useful for natural language and vision tasks and hold similar promise for the life sciences, provided they are suitably adapted to this new domain. We can see several uses for CellSAM that might be within reach of future work. First, given its generalization capabilities, it is likely that CellSAM has learned a general representation for the notion of “cells” used to query imaging data. These representations might serve as an interface between imaging data and other modalities (e.g., single-cell RNA Sequencing), provided there is suitable alignment between cellular representations for each domain ^83,84^. Second, much like what has occurred with natural images, we foresee that the integration of natural language labels in addition to cell-level labels might lead to vision-language models capable of generating human-like descriptors of cellular images with entity-level resolution ^46^. Third, the generalization capabilities may enable the standardization of cellular image analysis pipelines across all the life sciences. If the accuracy is sufficient, microbiologists and tissue biologists could use the same collection of foundation models for interpreting their imaging data even for challenging experiments ^85,86^.

While the work presented here highlights the potential foundation models hold for cellular image analysis, much work remains to be done for this future to manifest. Extension of this methodology to 3D imaging data is essential; recent work on memory-efficient attention kernels ^87^ will aid these efforts. Exploring how to enable foundation models to leverage the full information content of images (e.g., multiple stains, temporal information for movies, etc.) is an essential avenue of future work. Expanding the space of labeled data remains a priority - this includes images of perturbed cells and cells with more challenging morphologies (e.g., neurons). Data generated by pooled optical screens ^88^ may synergize well with the data needs of foundation models. Compute-efficient fine-tuning strategies must be developed to enable flexible adaptation to new image domains. Lastly, prompt engineering is a critical area of future work, as it is critical to maximizing model performance. The work we presented here can be thought of as prompt engineering, as we leverage CellFinder to produce bounding box prompts for SAM. As more challenging labeled datasets are incorporated, the nature of the “best” prompts will likely evolve. Finding the best prompts for these new data is a task that will likely fall on both the computer vision and life science communities.

## Supplementary Material

Supplement 1

## Data Availability

All datasets with test/training/validation splits are publicly available at https://cellsam.deepcell.org.
