# Supplementary material for: CellSAM: A Foundation Model for Cell Segmentation
Source: bioRxiv. 2025 Feb 13:2023.11.17.567630. Originally published 2023 Nov 20. Preprint. [Version 4] doi: 10.1101/2023.11.17.567630 (PMC10690226; doi:10.1101/2023.11.17.567630)
Supplement: Supplementary file 1 [file media-1.pdf]

Declarations

Code Availability

The code for CellSAM is publicly available at https://github.com/vanvalenlab/cellsam.

Data Availability

All datasets with test/training/validation splits are publicly available at https://cellsam.deepcell.org.

Author Contributions

UI, MM contributed equally to this project and have the right to list themselves first in bibliographic documents. RD, QL contributed equally to this project and have the right to list themselves second in bibliographic documents. UI, MM, YY, and DVV conceived the project; UI, MM, QL, YY, and DVV performed algorithm design for CellFinder and CellSAM; MM implemented the CellSAM architecture; UI, MM, and QL implemented CellFinder. UI and MM carried out the experiments and evaluations of the method. GG and PP provided input for developing CellFinder; QL and UI performed model benchmarking; QL and RD developed data pipelines, RD developed the computational infrastructure for model training; RD, EP, EP, MS, QL, CY, and EL performed data engineering; EL, CY, and UI performed CellSAM integration with bioimaging workflows. MM performed 3D CellSAM integration. AA, MA, CB performed annotations on images for human-human comparison. RB and DVV supervised the software engineering, DVV supervised the project.

Acknowledgements

We thank Leeat Keren, Noah Greenwald, Sam Cooper, Jan Funke, Uri Manor, Joe Horsman, Michael Baym, Paul Blainey, Ian Cheeseman, Manuel Leonetti, Neehar Kondapaneni, and Elijah Cole for valuable conversations and insightful feedback. We thank Felix Zhou for helping with the 3D segmentation. We also thank William Graf, Geneva Miller, and Kevin Yu, whose time in the Van Valen lab established the infrastructure and software tools that made this work possible. We thank Nader Khalil, Harper Carroll, Alec Fong, and the entire Brev.dev team for their support in establishing the computational infrastructure required for this work. We also thank Rosalind J. Xu and Jeffrey Moffitt for providing unpublished MERFISH data for the spatial transcriptomics workflow. We utilized images of the HeLa cell line in this research. Henrietta Lacks and the HeLa cell line established from her tumor cells without her knowledge or consent in 1951 has significantly contributed to scientific progress and advances in human health. We are grateful to Lacks, now deceased, and the Lacks family for their contributions to biomedical research. This work was supported by awards from the Shurl and Kay Curci Foundation (to DVV), the Rita Allen Foundation (to DVV), the Susan E. Riley Foundation (to DVV), the Pew-Stewart Cancer Scholars program (to DVV), the Gordon and Betty Moore Foundation (to DVV), the Schmidt Academy for Software Engineering (to SL), the Michael J. Fox Foundation through the Aligning Science Across Parkinson's consortium (to DVV), the Heritage Medical Research Institute (to DVV), the National Institutes of Health New Innovator program (DP2-GM149556) (to DVV), the National Institutes of Health HuBMAP consortium (OT2-OD033756) (to DVV), and the Howard Hughes Medical Institute Freeman Hrabowski Scholars program (to DVV).

National Institutes of Health (R01-MH123612A) (to PP). NIH/Ohio State University (R01-DC014498) (to PP). Chen Institute (to PP). The Emerald Foundation and Black in Cancer (to UI). Caltech Presidential Postdoctoral Fellowship Program (PPFP) (to UI).

Disclosures

David Van Valen is the scientific founder of Aizen Therapeutics and holds equity in the company. All other authors declare no competing interests.

A Dataset Construction

To train CellSAM, we combined ten separate datasets spanning a variety of modalities: TissueNet[28], DeepBacs[63], BriFiSeg[64], Cellpose[25,26], Omnipose[65,66], YeastNet[67], YeaZ[68], the 2018 Kaggle Data Science Bowl (DSB)[69], a collection of H&E datasets[70–76], and an internally collected dataset of phase microscopy images across eight mammalian cell lines (Phase400). The LIVECell[62] dataset was held out for zero-shot/few-shot testings. Our collective dataset included images across multiple imaging modalities (brightfield, phase contrast, fluorescence, and mass cytometry), imaging targets (histology sections, yeast, cell culture, bacteria, nuclei), length scales, and morphologies. During preprocessing, we reduced bright spots by linearly re-scaling the raw pixel intensities such that the 99.9 percentiles corresponded to 1.0. We then normalized each image with Contrast Limited Adaptive Histogram Equalization (CLAHE)[96] with a kernel size of 128 pixels. We treated nuclear and whole-cell channels as green and blue channels in an RGB image, respectively, and the red channel was always blank. We moved the green channel to blue for nuclear-only datasets (i.e., BriFiSeg and DSB) to keep the blue channel always occupied.

If available, we used pre-determined train/val/test splits for each dataset; otherwise, we introduced 80-10-10% data splits. For datasets with multiple fields of view of the same object set, we required all FOVs to belong to the same split. We deferred all duplicated samples to the train split for published datasets with a pre-existing data leak (detected by pixel-wise hashing). Our assembled dataset used a fixed image size of 512 by 512 pixels. Images shorter than 512 pixels on either axis were zero-padded up to 512. For images with more than 512 pixels on either axis, we tiled them to 512 by 512 pixels with a 25% overlap and filled the empty regions with zeros. Any cropped images without valid annotations were removed. We followed a widely used annotation scheme for labeling our masks, with zero representing the background and unique positive integers representing different objects. While this format precludes accurate segmentation of overlapping objects, labels of this kind were not present in the dataset we compiled. We filtered out invalid cell labels if the label contained disjoint regions or if the label had only a 1-pixel height or width. The cropped images with filtered annotations were used for training, validation, and testing. We conducted some additional processing for LIVECell[62]. We converted annotations from the COCO format to the same labeling format we used on the other datasets for consistency. We used Cellpose's[26] pre-processing function `livecell_ann_to_masks()` to remove overlapping regions. In addition, we noticed inconsistencies in ground truth labels as previously observed by the Cellpose team (see Fig 1.c in[26]). We thus manually inspected the LIVECell test split to divide the annotation quality into three classes - good, medium, and poor. We randomly selected images in the good split of the test set for the CellSAM few-shot learning task.

Statistics on our full dataset are in Table 1. To aid reproducibility, the dataset labels in Table 1 correspond to the labels present in our compiled dataset available at https://cellsam.deepcell.org.

B CellSAM Architecture.

We adapted Anchor DETR[61] for the object detector for CellSAM (CellFinder). This choice was motivated by Anchor DETR being non-maximum suppression (NMS)[97] free. NMS suppresses bounding boxes with a high amount of overlap to remove duplicate detections. While this works well for natural images, cellular images often have tightly clustered

Dataset	Test		Train		Val	
	Images	Objects	Images	Objects	Images	Objects
nuc_seg_dsb	56	2348	449	20485	57	2615
YeaZ	13	577	303	23971	2	49
Gendarme_BriFi	22	814	179	6570	23	909
YeastNet	15	576	120	6270	15	654
bact_fluor	23	5411	129	17128	6	697
bact_phase	145	18545	224	25791	22	1704
cellpose	68	7269	486	59187	54	6032
2b_brightfield_dataset	5	475	2	396	3	428
2b_fluorescence_dataset	5	475	2	354	3	428
2c_e_coli	15	1157	4	339	4	256
2d_1_SplineDist_dataset	10	659	22	407	10	148
2d_2_b_subtilis	10	659	70	3913	2	106
2e_e_coli	12	700	21	1104	4	273
s2_stardist	35	2766	120	6776	8	484
monuseg	7	3829	37	24140	7	2868
nuinsseg	1	25	9	334	2	53
tnbc	5	396	40	3297	5	413
cpm15	1	256	12	2060	2	604
cpm17	16	1771	32	3798	16	2065
monusac	21	3885	167	24019	21	3503
tissuenet_wholecell	330	132898	2576	987710	323	119623
tissuenet_nuclear	330	126638	2576	932590	323	114064
RAW264_ep_microscopy	13	589	100	7557	13	991
3T3_ep_microscopy	4	602	32	4105	5	625
HeLa_ep_microscopy	4	423	32	2890	4	363
HEK293_ep_microscopy	2	356	17	4432	3	527
CHO_ep_microscopy	4	985	29	6438	4	719
A549_ep_microscopy	7	515	60	5036	8	580
PC3_ep_microscopy	7	599	54	4793	7	592
HeLa-S3_ep_microscopy	13	3681	103	25476	13	2795
neurips_fixed	52	819306	1000	169445	101	141780

Table 1: Number of images and objects in each dataset split by test, train, and val.

objects, and NMS-based methods such as the R-CNN family[80,81] can suffer from a low recall in this setting. We replaced the Anchor DETR's ResNet[98] backbone with the vision transformer (ViT)[44] from the SAM model[52]; specifically, we used the base-sized ViT (ViT-B).

As the maximum number of cells per image is generally no more than 1000, we increased the number of queries q to 3500, 3.5 times the maximum number of cells, based on Fig. 12 in DETR[99], which provided an estimate of the number of queries needed for a DETR method to detect all objects. We used one pattern p for the Anchor generation as most objects in cellular detection are usually of similar scale.

Training CellFinder: We used a base learning rate of 10^{-4} for the Anchor DETR head and 10^{-5} for the SAM-ViT backbone. We used weight decay of 10^{-4} and clip norm of 0.1. We used AdamW[100] with a step-wise learning rate scheduler that drops the learning rate by 10% after 70% of the epochs. We trained CellFinder for 400 epochs with a batch size of 2 across 16 GPUs.

Finetuning CellSAM: After we trained CellFinder with the SAM-ViT backbone, the SAM-ViT output features were no longer aligned with the rest of the model (i.e., the prompt encoder and mask decoder). To close this distribution gap, we froze the SAM-ViT (such that it continues to function well with CellFinder) and trained the neck of the SAM model. . We trained this neck using ground-truth bounding boxes as inputs and segmentation masks of individual cells as targets. We used a learning rate of 10^{-4} and weight decay of 10^{-4} for this training. We also used AdamW[100] for this training and did not clip the gradient.

B.1 Inference

At inference, we followed the following workflow. First, the input was passed through the Anchor DETR fine-tuned ViT-B. This resulted in an embedding dimension of 768. This embedding was then passed as an input to two parts of CellSAM, 1) the trained Anchor DETR module (CellFinder) and 2) the fine-tuned neck, which is a 2D convolutional network reducing the embedding dimensionality further to 256. The bounding box outputs of CellFinder were then sent into the prompt encoder, resulting in the prompt embedding. The prompt embeddings and neck embedding were then passed to the mask decoder, which outputs pixel-wise probabilities for the cell and another IoU-based confidence value for the prediction as a whole. This results in a tensor of shape $N \times W \times H$, where N corresponds to the number of cells predicted. This tensor was processed with a sigmoid and a threshold operation, resulting in binarized images. Depending on the metric used, we either used this tensor directly with the N scores (specifically for computation of the COCO AP @ 0.5 IoU), or we computed the argmax over the cell dimension N to generate a tensor $W \times H$, where each pixel corresponded to a unique integer label for each cell.

Thresholding: Given CellSAM's model architecture, we had three different thresholds at inference time. First, we had a threshold on the bounding boxes generated by CellFinder, which we set to 0.4 across all datasets. We dynamically adjust this threshold by clustering the confidence values. We perform k-means clustering ($k = 2$) the box-confidences of all cells for a given images. We then compute the mean of both clusters (T_μ, i.e. the seperation between likely non-cells and cells. We then adjust the final bounding box threshold as $T_{box} = \frac{2}{3} * 0.4 + \frac{1}{3} * T_\mu$. The resulting boxes were passed through the Mask Decoder, we had an overall mask score outputted by the IoU prediction head of the Mask Decoder, which we set to 0.5. Lastly, we thresholded the mask decoder output after applying the sigmoid function to each pixel, which we set at 0.5.

CellSAM Postprocessing: We used the same postprocessing steps that are used by SAM[52]. This consisted of hole filling and island removal for each predicted cell.

Inference time: Here we show the inference time as a function of the number of cells.

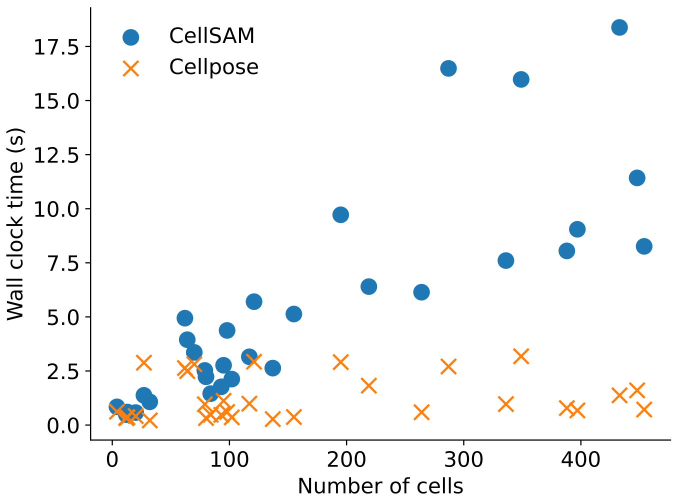

Fig. S1: Inference time for the CellSAMand Cellpose.

B.1.1 Model Implementation and Training

CellSAM was implemented in Pytorch[101]. For CellFinder we modified the official Anchor DETR repo[1]. For CellSAM, we modified the official Segment Anything repo[2]. We used Pytorch Lightning[102] to scale the training. Prototyping was done using NVIDIA's RTX 4090. We used machines with either NVIDIA A6000s or A100s (40GB and 80GB versions) for the experiments in the paper. The overall training time was 6d 5h 33m 20s for training Cellfinder and 15h 33m 57s for subsequent finetuning.

B.2 3D integration

We use u-segment3D[89] to generate 3D cellular segmentations. We first segment each 2D slice using the CellSAMgeneral model. We then remove small masks and fill holes using Cellposes' postprocessing. We then use the u-segment3D[89] indirect method to fuse the 2D segmentations to 3D.

C Benchmarking

We benchmarked the performance of CellSAM models against Cellpose[25,26] trained on our compiled datasets.

C.1 Cellpose Model Training.

We utilized the command provided by the Cellpose developers to train our custom Cellpose models. Using Cellpose library version 3.0.11, we trained both specialist and generalist models from scratch[26].

```
python -m cellpose --train --train_size --use_gpu --dir {} --test_dir {}
--img_filter _img --mask_filter _masks --pretrained_model None --chan 3 -chan2 2
```

We kept all the hyper-parameters untouched. We used the SGD optimizer with a weight decay of 10^{-5} and a batch size of 8. We trained each model for 500 epochs with a base learning rate of 0.2. We used the default learning rate scheduler. The learning rate increased linearly from 0 to 0.2 over the first ten epochs, then decreased by a factor of 2 every 10 epochs after the 400th epoch. We trained each model on a single NVIDIA A6000 GPU. In total, we trained ten specialist models and one generalist model. When evaluating the performance of the cellpose model, we followed the implementation of the cyto3 model evaluating pipeline. We first estimated the cell diameters per image using a trained size model. Then, we predicted the segmentation masks.

C.2 Metrics

We used the Metrics package in the Cellpose library[25,26]. Predictions that match the ground truth labels (determined by a mask IoU ≥ 0.5) are true positives (TP), predictions with no matching ground truth labels are false positives (FP), and ground truth labels without a valid match are false negatives (FN). For human-to-human comparisons, we used a lower threshold of 0.3 to reflect the different styles of different annotators. We computed the recall, precision, and F1 scores using the following formulas:

[1] https://github.com/megvii-research/AnchorDETR
[2] https://github.com/facebookresearch/segment-anything

- Recall: $\text{recall} = \frac{\text{TP}}{\text{TP}+\text{FN}}$.
- Precision: $\text{precision} = \frac{\text{TP}}{\text{TP}+\text{FP}}$.
- F1: $F_1 = \frac{2\times\text{precision}\times\text{recall}}{\text{precision}+\text{recall}}$.

We also used the COCO evaluation metrics[79] during CellFinder's development. The COCO metrics are a widely used benchmark for assessing the object-level quality of object detection and instance segmentation methods. These metrics report Average Precision (AP), the area under the Precision-Recall curve for a given object class. In our case, we only had a single object class: cells. The AP is computed for different IoU thresholds, ranging from 0.5 to 0.95, with a step size of 0.05. We report the mean AP across all IoU thresholds, denoted as `mAP`, as well as the AP at IoU=0.5, denoted as `AP50`, to quantify CellFinder's performance. Because the object density is much higher in cellular images than in natural images, we modified the limit for the maximum number of detections from 100 to 10,000. We also fed the actual confidence score per binary prediction of the CellSAM model to the COCO evaluator. For the Cellpose models, we used a fixed confidence score of 1.0.

D Hyperparameters

For training, there are two sets of parameters. The first is for training CellFinder , which we trained for 400 epochs with a batch size of 4 (primarily constrained by GPU memory) across 8-16 GPUs per model (either A100s or H100s). We used the AdamW optimizer with a learning rate of 10^{-5} for the backbone and 10^{-4} for the remainder of the model. We set weight decay 10^{-4} and dropout to 0.1. After 240 epochs, we reduced the learning rate by 0.1. The full configuration file can be found in the Github repository.

The second set of parameters is fine-tuning CellSAM which we trained for one epoch. We used 3500 query positions and used the default configuration available in the GitHub repository for the rest of the parameters.

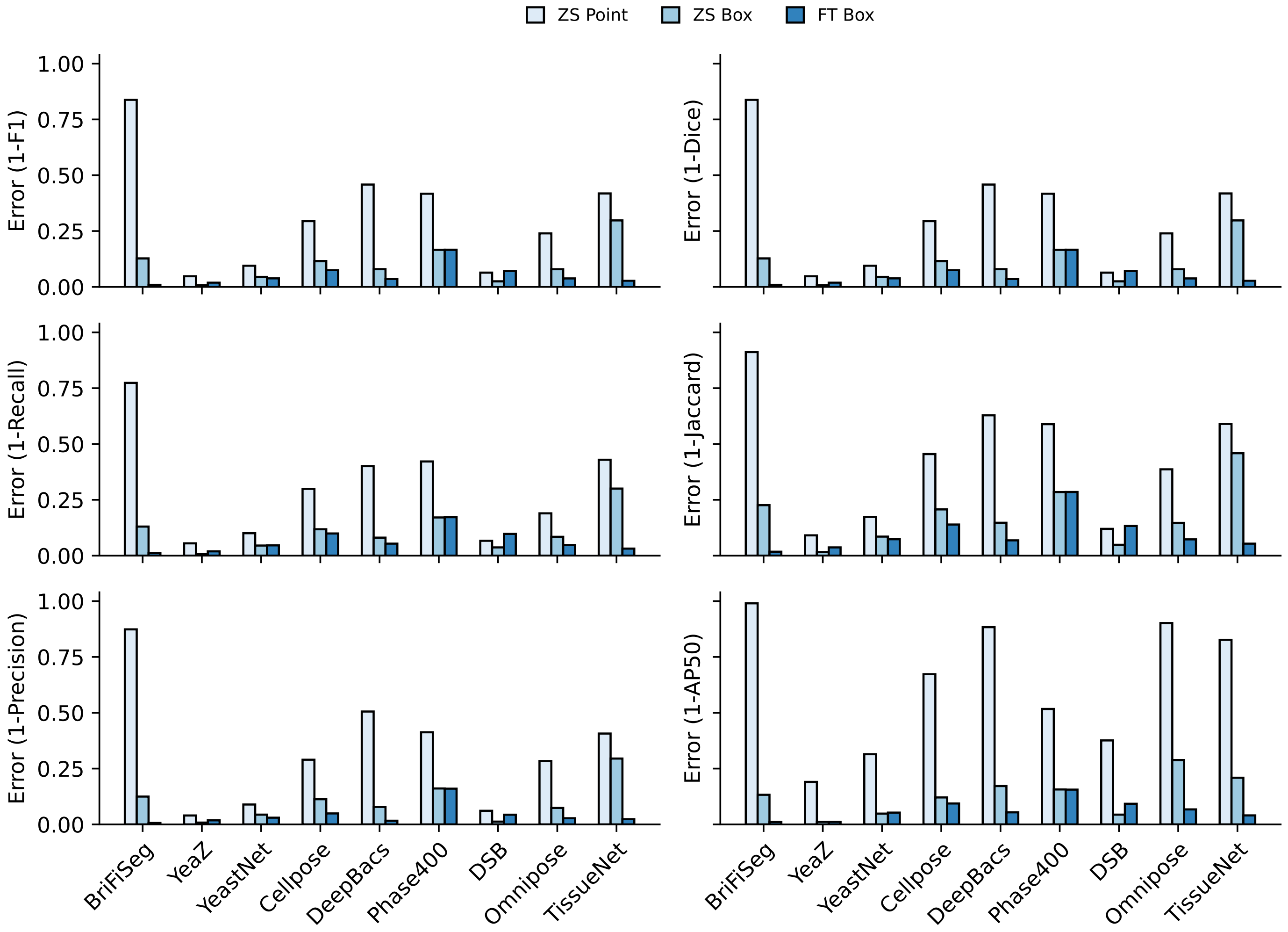

Fig. S2: **Per dataset performance comparing zero-shot (ZS Point) point prompting, zero-shot box prompting (ZS Box), and fine-tuned box (FT Box) prompting** across a suite of metrics from the DeepCell package, and additionally, we included the AP50 from the COCO metrics. We showed the error rate (1-metric) on these bar plots. We demonstrated CellSAM-specialist and CellSAM-generalist superior performance across multiple datasets and multiple evaluation metrics.

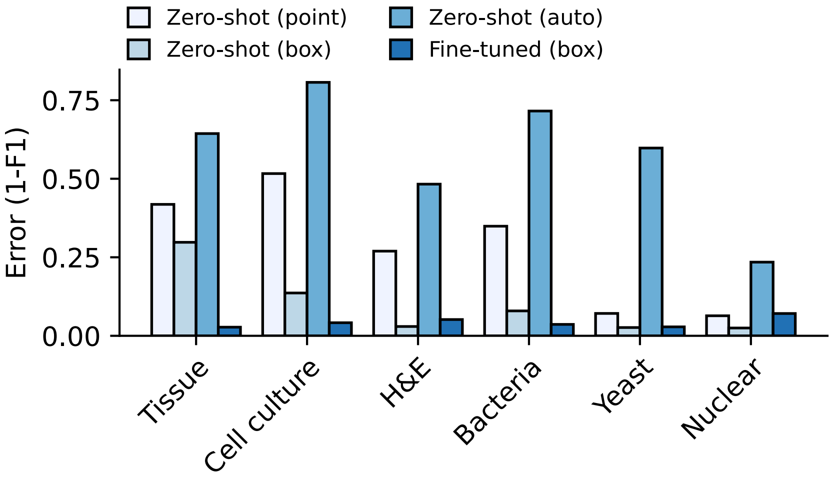

Fig. S3: **Preliminary prompting analysis** comparing point prompts, bounding box prompts, and SAM's auto prompting (a uniform grid of points). We used the ground truth cellular masks to generate both the point and bounding box prompts (serving as a theoretical upper bound on performance). We used the F1 error (1-F1 score) for the metric of comparison. Additionally, we used SAM with no additional training to asses zero-shot performance and compared that to a fine-tuned SAM. Here we see the best performance was achieved using bounding box prompts with a fine-tuned model.

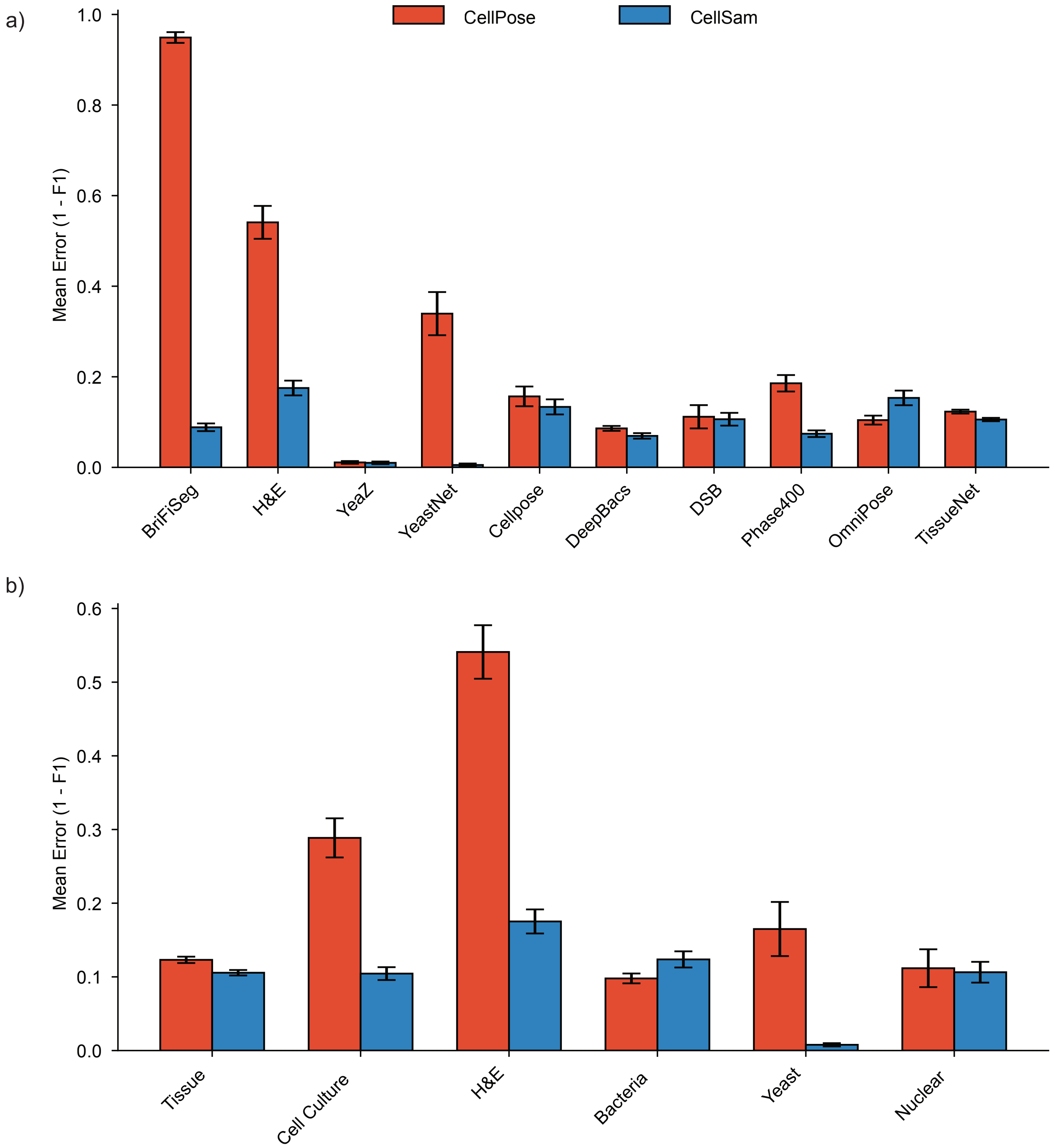

Fig. S4: **CellSAM-generalist Comapred to CellPose generalist model (Cyto3)**. Here, we compare CellSAM-generalist performance to Cellpose's Cyto3 model. a) examines the per dataset performance of these two models. b) examples the performance across the various data types.

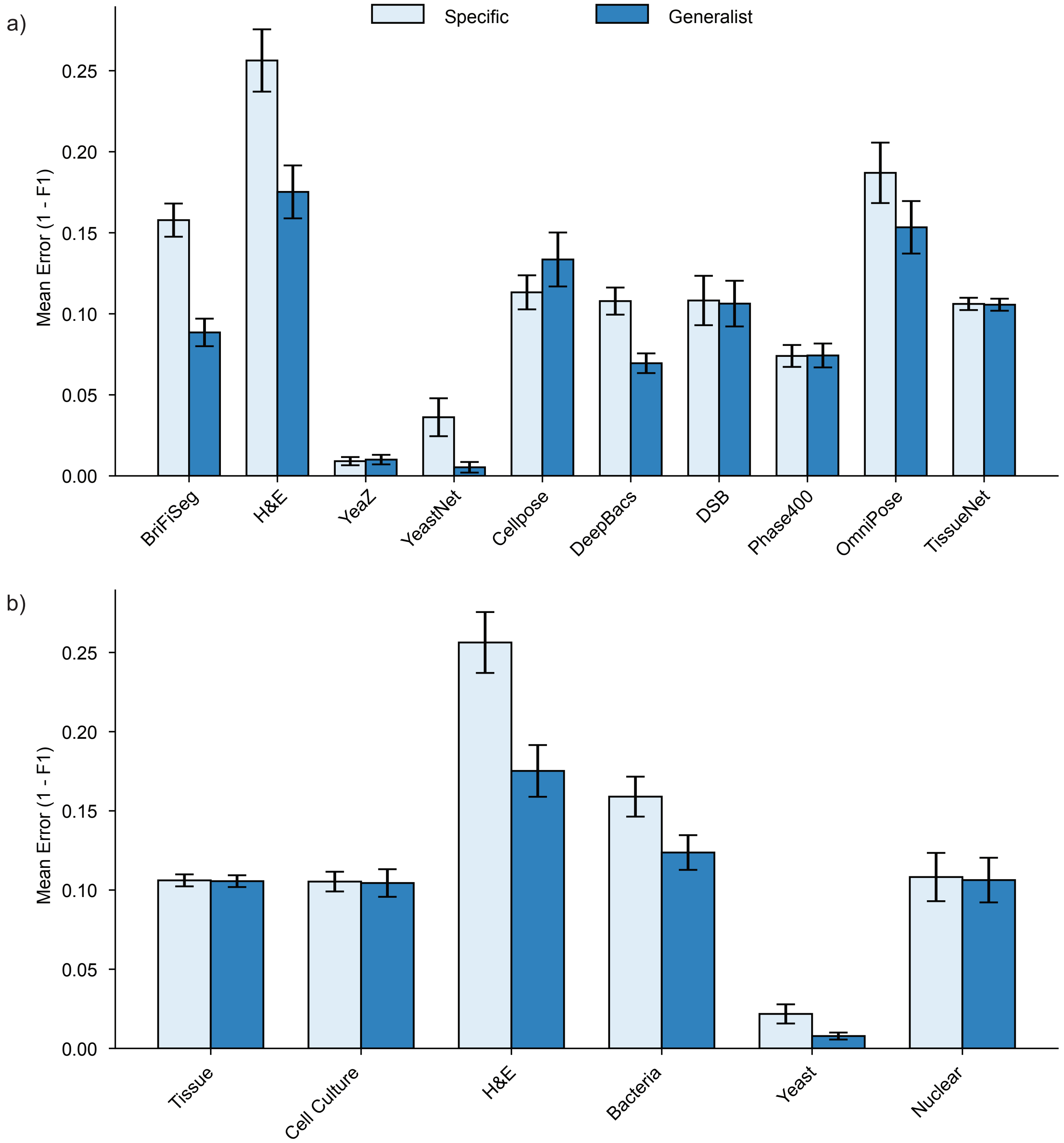

Fig. S5: **CellSAM-generalist vs CellSAM-specialist** We compare the performance of CellSAM when trained on individual datasets (specific) to when CellSAM is trained on all of the data (generalist). We see that CellSAM-generalist performs better across data types and datasets.

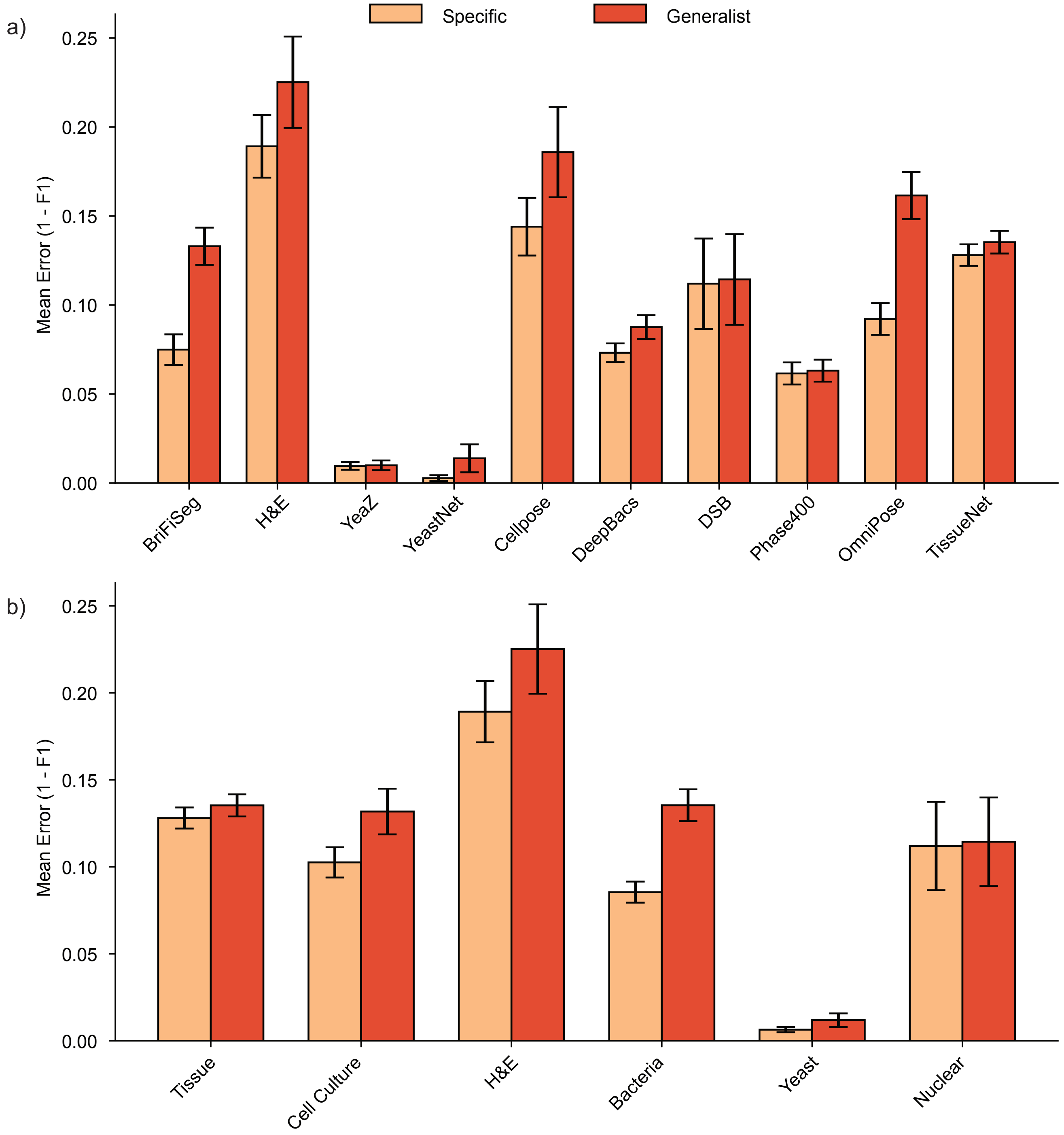

Fig. S6: **CellPose-generalist vs CellPose-specialist**. Here, we compare the performance of Cellpse when trained on individual datasets (specific) to when Cellpse is trained on all the data (generalist). Cellpose consistently performs better when trained on a specific datset but does not perform well when trained across multiple datasets.

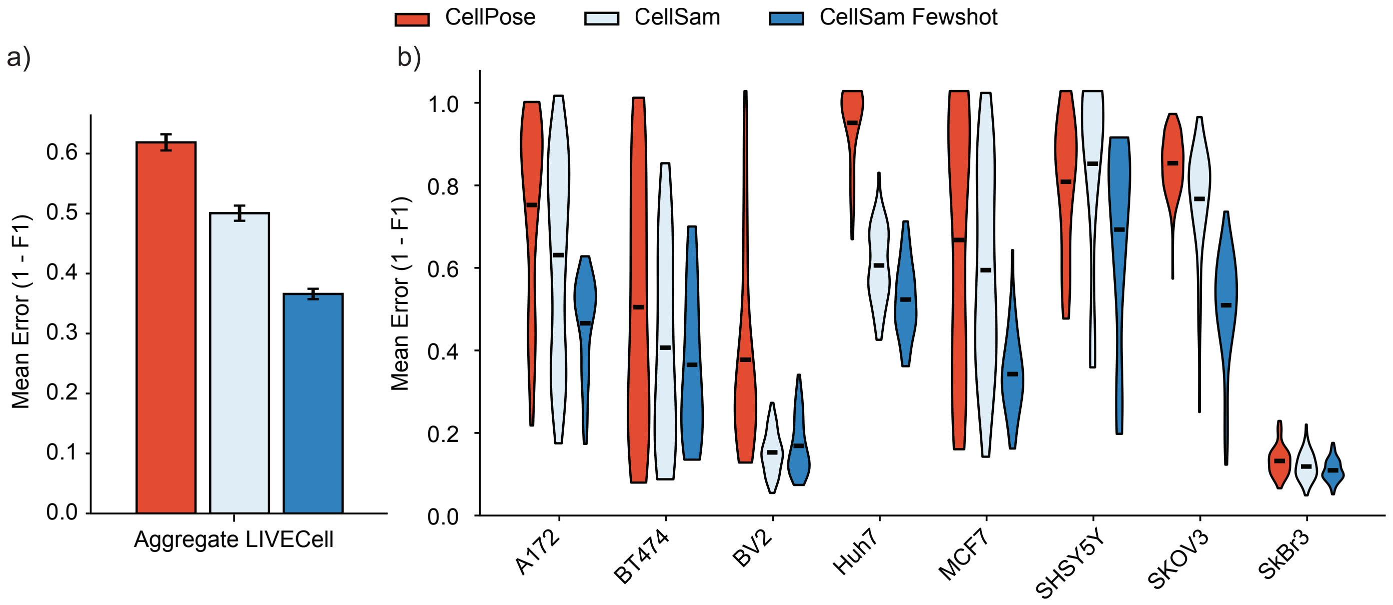

Fig. S7: a) Zero-shot performance of CellSAM-generalist and Cellpose-general on the LIVECell dataset. Here, we show significant improvement of CellSAM over Cellpose on an unseen dataset (from 0.13 to 0.40 in F1). b) CellSAM-generalist performance stratified by cell line. We analyzed both zero-shot and few-shot (10 samples per cell line) performance. We saw that few-shot improves CellSAM-generalist on LIVECell for most cell lines.

A172

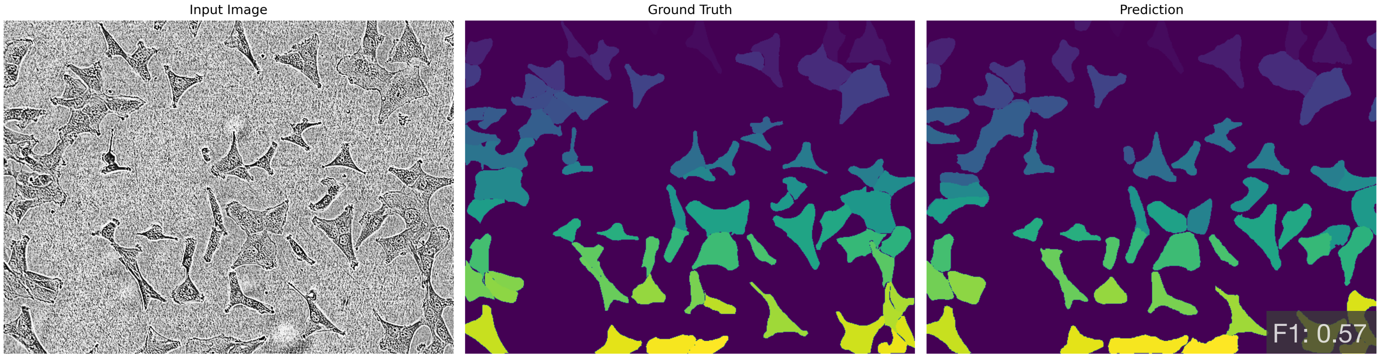

BT474

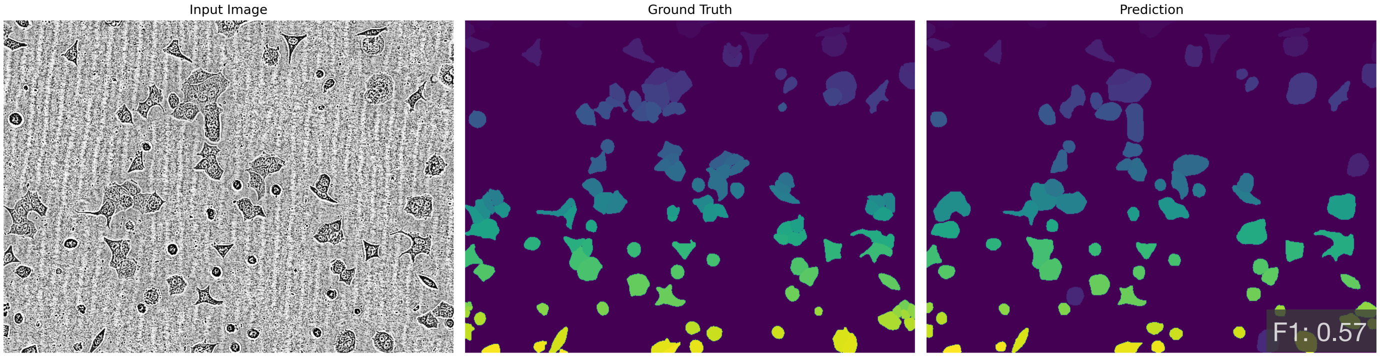

BV2

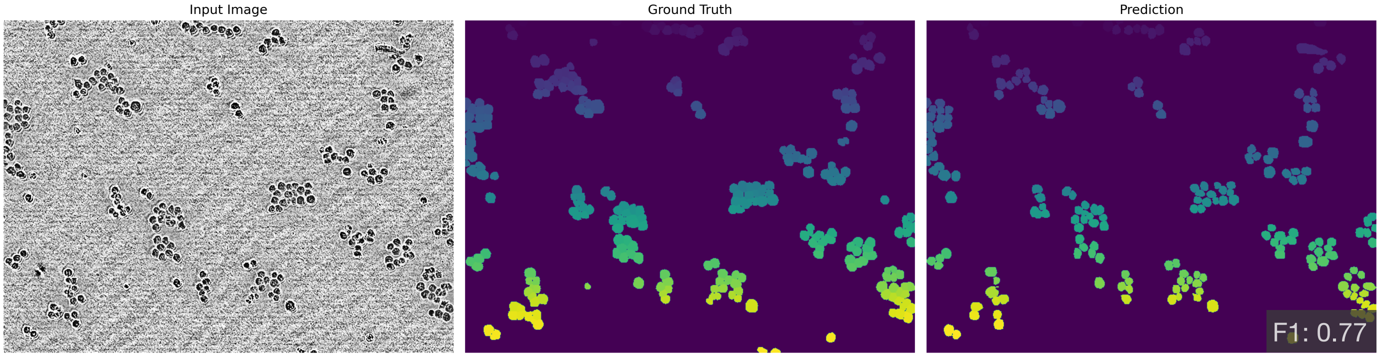

Huh7

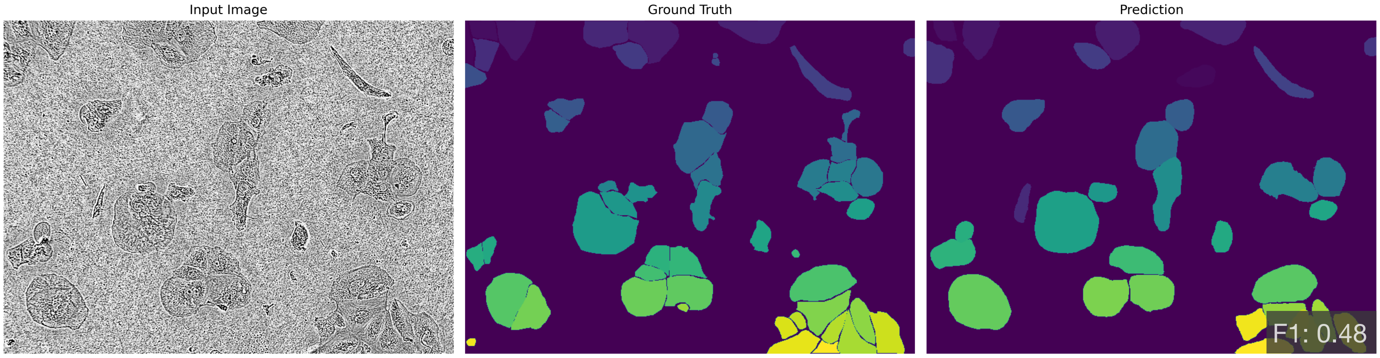

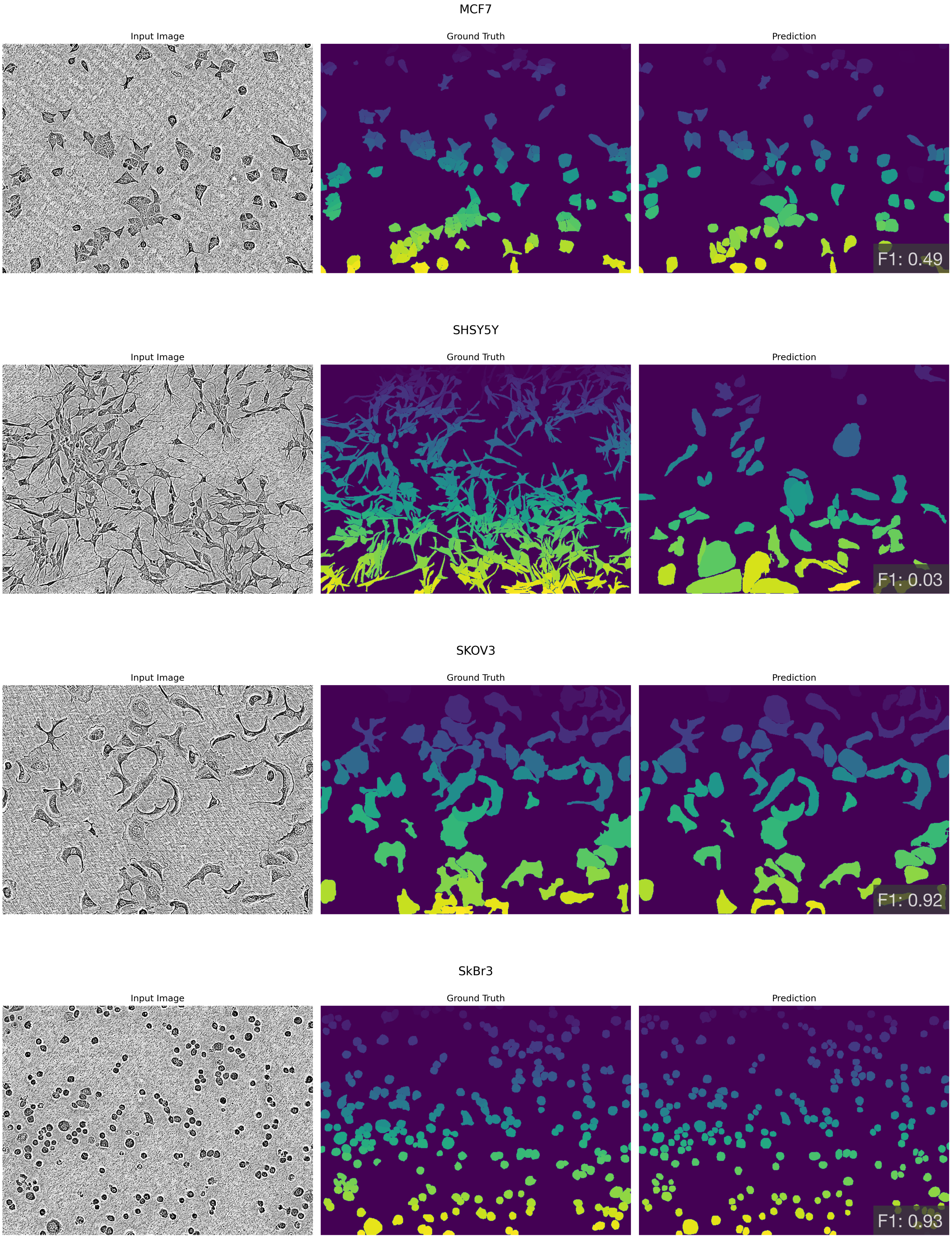

Fig. S8: Representative qualitative results of CellSAM-generalist 10-shot performance on the LIVECell dataset, one panel for each cell line.
