# Supplementary material for: A Foundation Model for Cell Segmentation
Source: bioRxiv. 2024 Mar 7:2023.11.17.567630. Originally published 2023 Nov 20. Preprint. [Version 3] doi: 10.1101/2023.11.17.567630 (PMC10690226; doi:10.1101/2023.11.17.567630)
Supplement: Supplementary file 1 [file media-1.pdf]

A Dataset Construction

To train CellSAM, we combined ten separate datasets spanning a variety of modalities: TissueNet[27], DeepBacs[59], BriFiSeg[60], Cellpose[24,25], Omnipose[61,62], YeastNet[63], YeaZ[64], the 2018 Kaggle Data Science Bowl (DSB)[65], a collection of H&E datasets[66–72], and an internally collected dataset of phase microscopy images across eight mammalian cell lines (Phase400). The LIVECell[58] dataset was held out for zero-shot/few-shot testings. Our collective dataset included images across multiple imaging modalities (brightfield, phase contrast, fluorescence, and mass cytometry), imaging targets (histology sections, yeast, cell culture, bacteria, nuclei), length scales, and morphologies. During preprocessing, we reduced bright spots by linearly re-scaling the raw pixel intensities such that the 99.9 percentiles corresponded to 1.0. We then normalized each image with Contrast Limited Adaptive Histogram Equalization (CLAHE)[89] with a kernel size of 128 pixels. We treated nuclear and whole-cell channels as green and blue channels in an RGB image, respectively, and the red channel was always blank. We moved the green channel to blue for nuclear-only datasets (i.e., BriFiSeg and DSB) to keep the blue channel always non-empty.

If available, we used pre-determined train/val/test splits for each dataset; otherwise, we introduced 80-10-10% data splits. For datasets with multiple fields of view of the same object set, we required all FOVs to belong to the same split. We deferred all duplicated samples to the train split for published datasets with a pre-existing data leak (detected by pixel-wise hashing). Our assembled dataset used a fixed image size of 512 by 512 pixels. Images shorter than 512 pixels on either axis were zero-padded up to 512. For images with more than 512 pixels on either axis, we tiled them to 512 by 512 pixels with a 25% overlap and filled the empty regions with zeros. Any cropped images without valid annotations were removed. We followed a widely used annotation scheme for labeling our masks, with zero representing the background and unique positive integers representing different objects. While this format precludes accurate segmentation of overlapping objects, labels of this kind were not present in the dataset we compiled. We filtered out invalid cell labels if the label contained disjoint regions or if the label had only a 1-pixel height or width. The cropped images with filtered annotations were used for training, validation, and testing. We conducted some additional processing for LIVECell[58]. We converted annotations from the COCO format to the same labeling format we used on the other datasets for consistency. We used Cellpose's[25] pre-processing function `livecell_ann_to_masks()` to remove overlapping regions. In addition, we noticed inconsistencies in ground truth labels as previously observed by the Cellpose team (see Fig 1.c in[25]). We thus manually inspected the LIVECell test split to divide the annotation quality into three classes - good, medium, and poor. We randomly selected images in the good split of the validation set for the CellSAM few-shot learning task.

B CellSAM Architecture.

We adapted Anchor DETR[57] for the object detector for CellSAM (CellFinder). This choice was motivated by Anchor DETR being non-maximum suppression (NMS)[90] free. NMS suppresses bounding boxes with a high amount of overlap to remove duplicate detections. While this works well for natural images, cellular images often have tightly clustered objects, and NMS-based methods such as the R-CNN family[75,76] can suffer from a low recall in this setting. We replaced

the Anchor DETR's ResNet[91] backbone with the vision transformer (ViT)[42] from the SAM model[48]; specifically, we used the base-sized ViT (ViT-B).

As the maximum number of cells per image is generally no more than 1000, we increased the number of queries q to 3500, 3.5 times the maximum number of cells, based on Fig. 12 in DETR[92], which provided an estimate of the number of queries needed for a DETR method to detect all objects. We used one pattern p for the Anchor generation as most objects in cellular detection are usually of similar scale.

Training CellFinder We used a base learning rate of 10^{-4} for the Anchor DETR head and 10^{-5} for the SAM-ViT backbone. We used weight decay of 10^{-4} and clip norm of 0.1. We used AdamW[93] with a step-wise learning rate scheduler that drops the learning rate by 10% after 70% of the epochs. We trained CellFinder for 500 epochs (1000 for smaller datasets) with a batch size of 2 across 16 GPUs.

Finetuning CellSAM After we trained CellFinder with the SAM-ViT backbone, the SAM-ViT output features were no longer aligned with the rest of the model (i.e., the prompt encoder and mask decoder). To close this distribution gap, we froze the SAM-ViT (such that it continues to function well with CellFinder) and trained the neck of the SAM model. The neck is a 2D-convolutional neural network that embeds the ViT features (e.g., 768 for SAM-ViT-B) to a 256-dimensional embedding that is then used as the primary feature vector for the rest of the model (prompt embedding and mask decoder). We trained this neck using ground-truth bounding boxes as inputs and segmentation masks of individual cells as targets. We used a learning rate of 10^{-4} and weight decay of 10^{-4} for this training. We also used AdamW[93] for this training and did not clip the gradient.

B.1 Inference

At inference, we followed the following workflow. First, the input was passed through the Anchor DETR fine-tuned ViT-B. This resulted in an embedding dimension of 768. This embedding was then passed as an input to two parts of CellSAM, 1) the trained Anchor DETR module (CellFinder) and 2) the fine-tuned neck, which is a 2D convolutional network reducing the embedding dimensionality further to 256. The bounding box outputs of CellFinder were then sent into the prompt encoder, resulting in the prompt embedding. The prompt embeddings and neck embedding were then passed to the mask decoder, which outputs pixel-wise probabilities for the cell and another IoU-based confidence value for the prediction as a whole. This results in a tensor of shape $N \times W \times H$, where N corresponds to the number of cells predicted. This tensor was processed with a sigmoid and a threshold operation, resulting in binarized images. Depending on the metric used, we either used this tensor directly with the N scores (specifically for computation of the COCO AP @ 0.5 IoU), or we computed the argmax over the cell dimension N to generate a tensor $W \times H$, where each pixel corresponded to a unique integer label for each cell.

Thresholding. Given CellSAM's model architecture, we had three different thresholds at inference time. First, we had a threshold on the bounding boxes generated by CellFinder, which we set to 0.4 across all datasets. After the boxes were passed through the Mask Decoder, we had an overall mask score outputted by the IoU prediction head of the Mask Decoder, which we set to 0.5. Lastly, we thresholded the mask decoder output after applying the sigmoid function to each pixel, which we set at 0.5.

CellSAM Postprocessing. We used the same postprocessing steps that are used by SAM[48]. This consisted of hole filling and island removal for each predicted cell.

B.1.1 Model Implementation and Training

CellSAM was implemented in Pytorch[94]. For CellFinder we modified the official Anchor DETR repo[1]. For CellSAM, we modified the official Segment Anything repo[2]. We used Pytorch Lightning[95] to scale the training. Prototyping was done using NVIDIA's RTX 4090. We used machines with either NVIDIA A6000s or A100s (40GB and 80GB versions) for the experiments in the paper.

C Benchmarking

We benchmarked the performance of CellSAM models against Cellpose[24,25] trained on our compiled datasets.

C.1 Cellpose Model Training.

We followed the hyper-parameters described in the original paper[25] to train specialist and generalist Cellpose models from scratch. We used the SGD optimizer with a weight decay of 10^{-4} and a batch size of eight. We trained each model for 300 epochs with a base learning rate of 0.1. We used the default learning rate scheduler in Cellpose 2.2.3. The learning rate increased linearly from 0 to 0.1 over the first ten epochs, then decreased by a factor of two every ten epochs after the 200th epoch. We trained each model on a single NVIDIA A6000 GPU. In total, we trained ten specialist models and one generalist model.

C.2 Metrics

We used the Metrics package in the DeepCell library[27,32], which is a set of tools for object-level evaluation of cell segmentations. Predictions that match the ground truth labels (determined by a mask IoU ≥ 0.6) are true positives (TP), predictions with no matching ground truth labels are false positives (FP), and ground truth labels without a valid match are false negatives. We computed the recall, precision, and F1 scores using the following formulas:

- Recall: $\text{recall} = \frac{\text{TP}}{\text{TP+FN}}$.
- Precision: $\text{precision} = \frac{\text{TP}}{\text{TP+FP}}$.
- F1: $F_1 = \frac{2\times\text{precision}\times\text{recall}}{\text{precision}+\text{recall}}$.

Details of the implementation of these metrics are described in prior work[32].

We also used the COCO evaluation metrics[74] during CellFinder's development. The COCO metrics are a widely used benchmark for assessing the object-level quality of object detection and instance segmentation methods. These metrics report Average Precision (AP), the area under the Precision-Recall curve for a given object class. In our case, we only had a single object class: cells. The AP is computed for different IoU thresholds, ranging from 0.5 to 0.95, with a step size of 0.05. We report the mean AP across all IoU thresholds, denoted as `mAP`, as well as the AP at IoU=0.5, denoted

[1] https://github.com/megvii-research/AnchorDETR
[2] https://github.com/facebookresearch/segment-anything

as `AP50`, to quantify CellFinder's performance. Because the object density is much higher in cellular images than in natural images, we modified the limit for the maximum number of detections from 100 to 10,000. We also fed the actual confidence score per binary prediction of the CellSAM model to the COCO evaluator. For the Cellpose models, we used a fixed confidence score of 1.0.

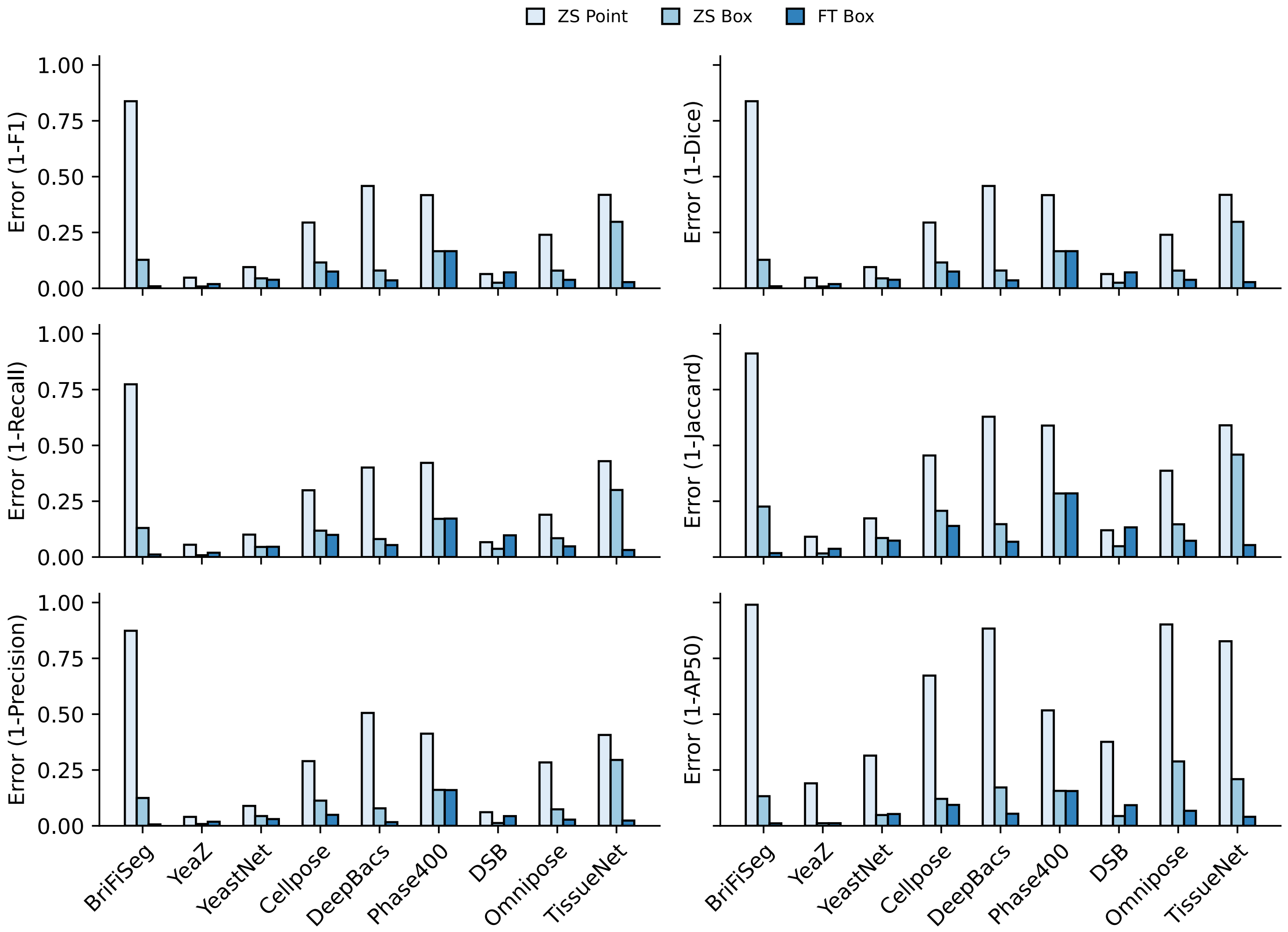

Fig. S1: **Per dataset performance comparing zero-shot point prompting, zero-shot box prompting, and fine-tuned box prompting** across a suite of metrics from the DeepCell package, and additionally, we included the AP50 from the COCO metrics. We showed the error rate (1-metric) on these bar plots. We demonstrated CellSAM-specificand CellSAM-generalsuperior performance across multiple datasets and multiple evaluation metrics.

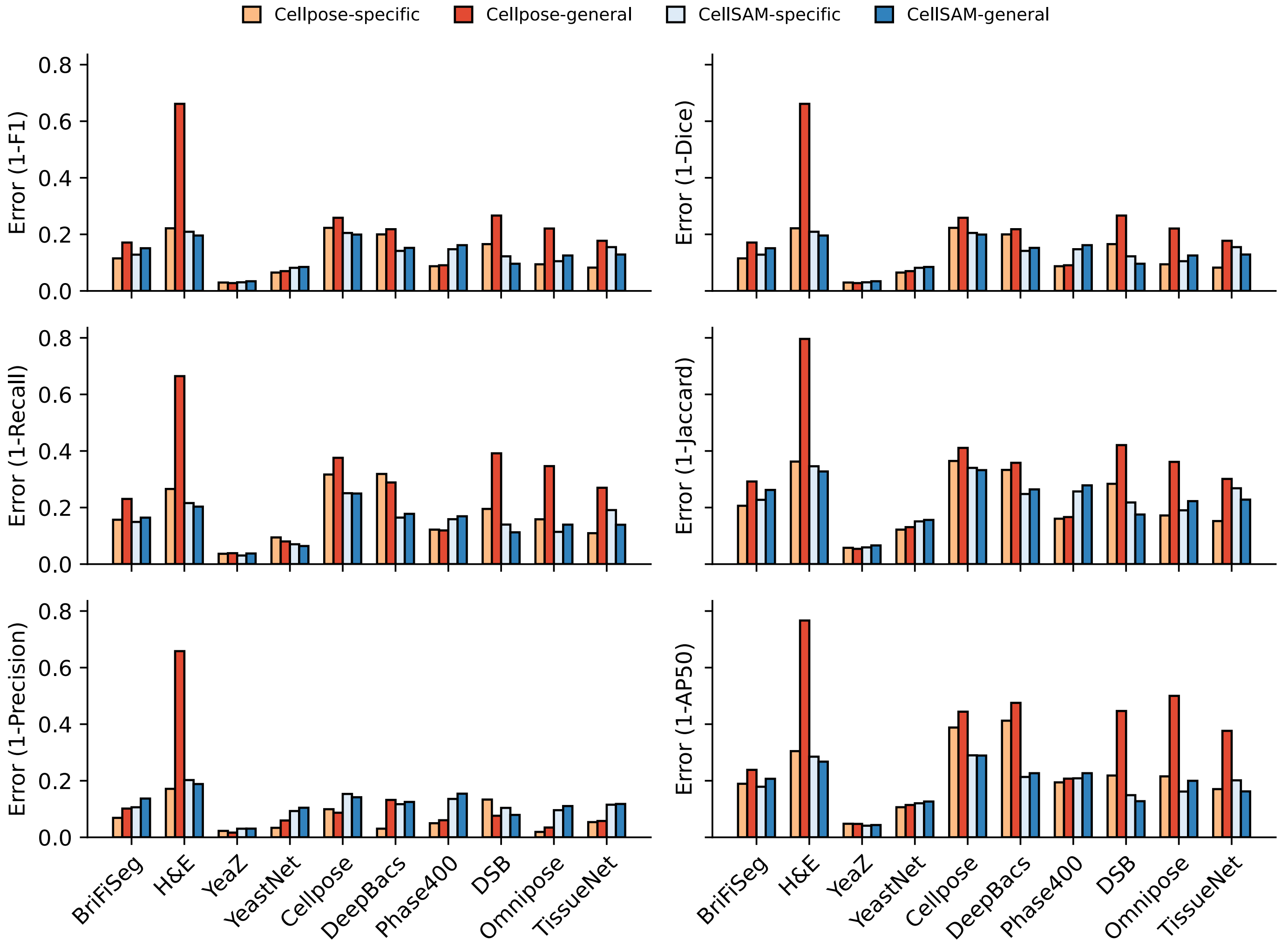

Fig. S2: **Per dataset performance** across a suite of metrics from the DeepCell package, and additionally, we included the AP50 from the COCO metrics. We showed the error rate (1-metric) on these bar plots. We demonstrated CellSAM-specificand CellSAM-generalsuperior performance across multiple datasets and evaluation metrics.

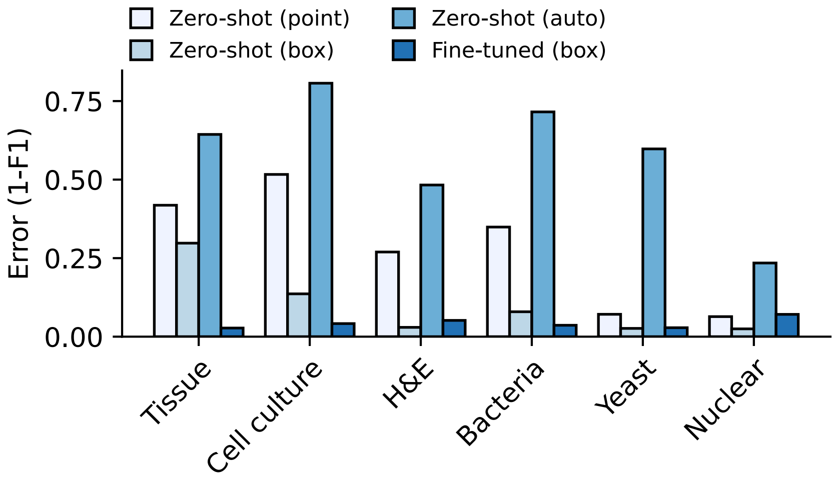

Fig. S3: Zero-shot (ZS) and fine-tuned mask generation error (1- F1 score) for SAM when using point and bounding box prompts. All prompting in this figure was done with ground truth prompts. The best performance was achieved with bounding box prompts and fine-tuning.

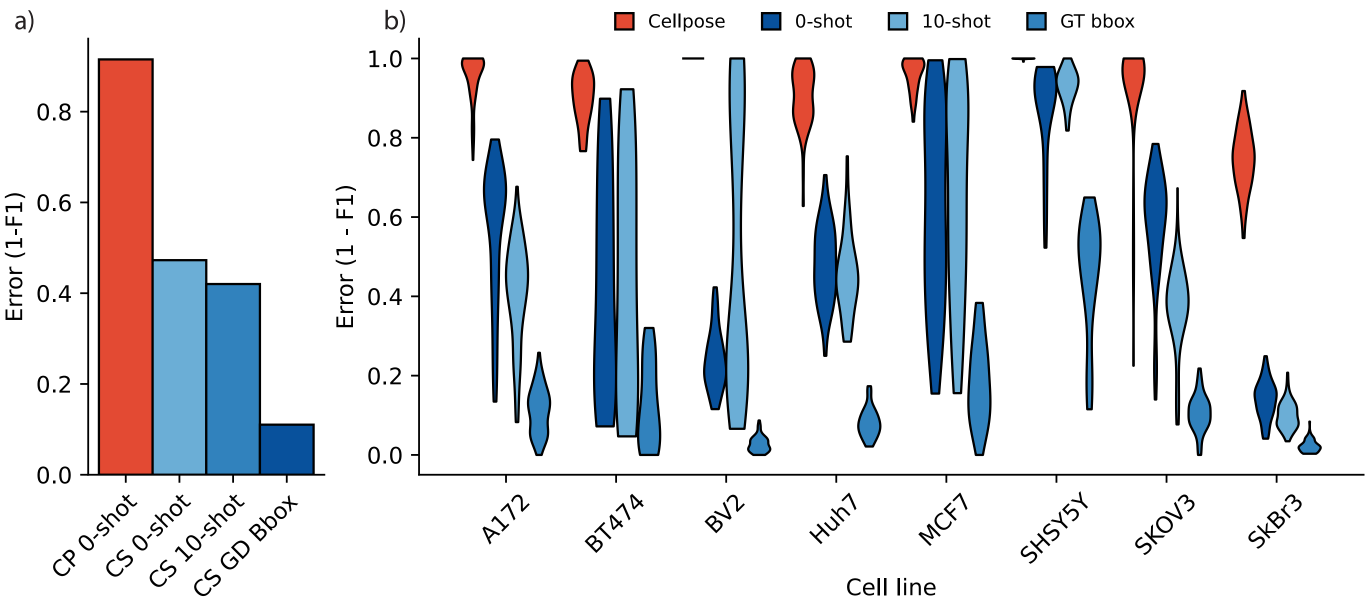

Fig. S4: a) Zero-shot performance of CellSAM-general and Cellpose-general on the LIVECell dataset. Here, we showed greater than 3x segmentation performance on an unseen dataset (from 0.13 to 0.40 in F1). b) CellSAM-general performance stratified by cell line. We analyzed both zero-shot and few-shot performance. We saw that few-shot improves CellSAM-general on LIVECell for some cell lines.

A172

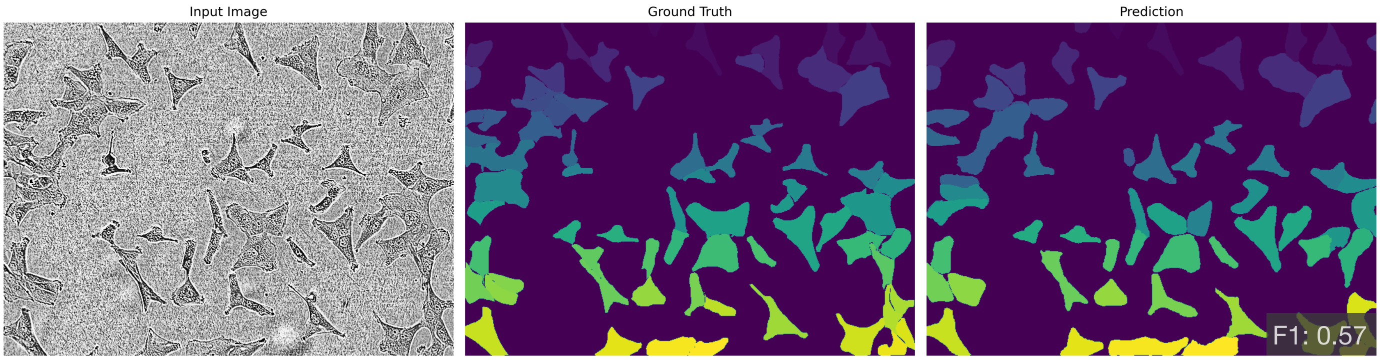

BT474

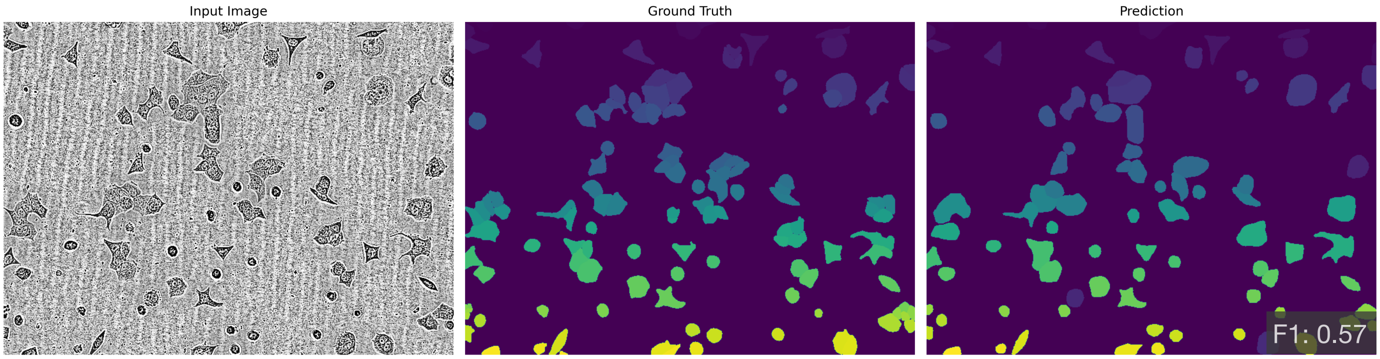

BV2

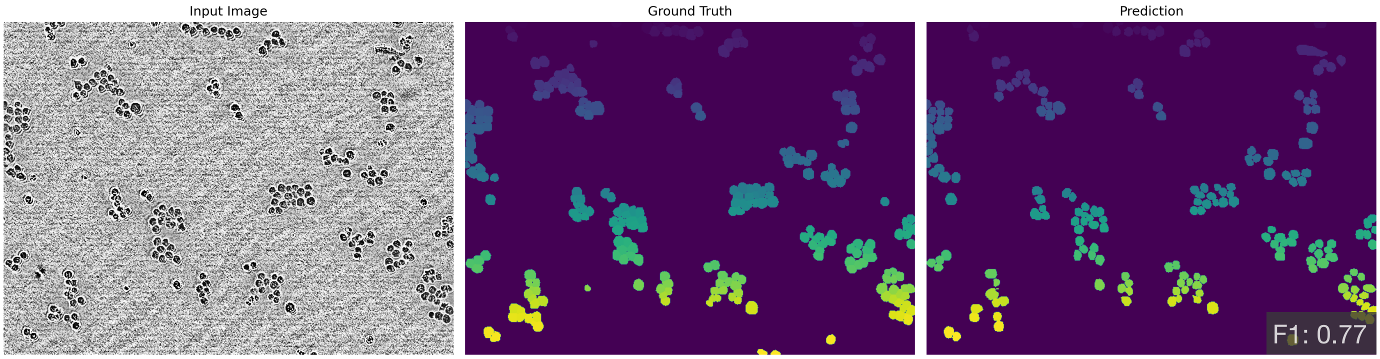

Huh7

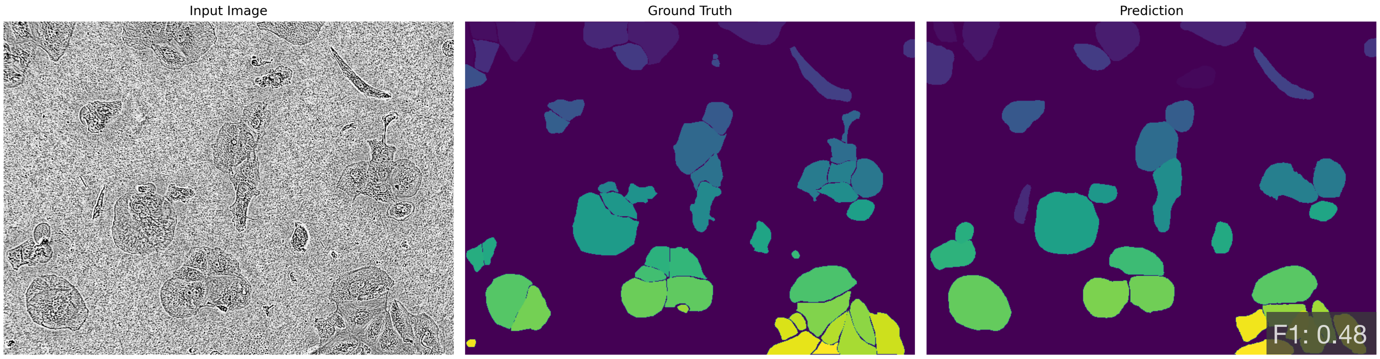

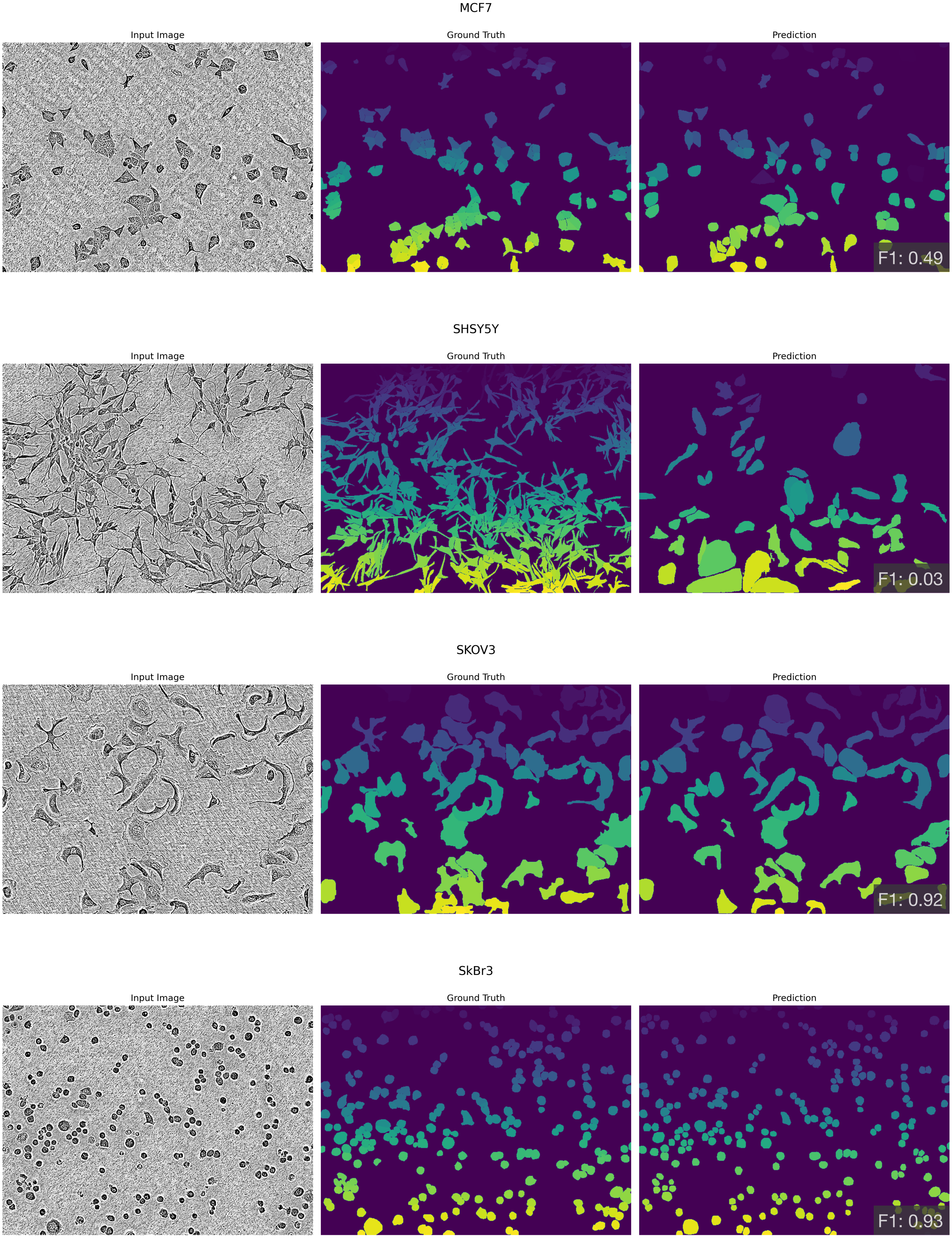

Fig. S5: Representative qualitative results of CellSAM-general 10-shot performance on the LIVECell dataset, one panel for each cell line.
